# The impacts of drift and selection on genomic evolution in insects

**DOI:** 10.7717/peerj.3241

**Published:** 2017-04-27

**Authors:** K. Jun Tong, Sebastián Duchêne, Nathan Lo, Simon Y.W. Ho

**Affiliations:** 1 School of Life and Environmental Sciences, University of Sydney, Sydney, New South Wales, Australia; 2 Centre for Systems Genomics, University of Melbourne, Melbourne, Victoria, Australia

**Keywords:** Mutation, Genomic pacemakers, Molecular evolution, Neutral theory, Insect phylogenomics

## Abstract

Genomes evolve through a combination of mutation, drift, and selection, all of which act heterogeneously across genes and lineages. This leads to differences in branch-length patterns among gene trees. Genes that yield trees with the same branch-length patterns can be grouped together into clusters. Here, we propose a novel phylogenetic approach to explain the factors that influence the number and distribution of these gene-tree clusters. We apply our method to a genomic dataset from insects, an ancient and diverse group of organisms. We find some evidence that when drift is the dominant evolutionary process, each cluster tends to contain a large number of fast-evolving genes. In contrast, strong negative selection leads to many distinct clusters, each of which contains only a few slow-evolving genes. Our work, although preliminary in nature, illustrates the use of phylogenetic methods to shed light on the factors driving rate variation in genomic evolution.

## Introduction

Molecular evolution proceeds by the fixation of mutations, a process that balances stochastic drift against natural selection. The relative importance of these two forces depends on population size (Ohta, 1992) and on the distribution of fitness effects of new mutations (Eyre-Walker & Keightley, 2007). When mutations have neither a beneficial nor detrimental impact on fitness, their fate is determined entirely by the stochastic process of genetic drift (Kimura, 1968). In contrast, purifying selection removes deleterious mutations over time. Selection is more efficient in large populations, where even small differences in selection coefficients can substantially change the relative probability of any particular mutation becoming fixed (Ohta, 1992). In small populations, mutations with small fitness effects behave similarly to neutral mutations, so drift tends to be more important. 

**Figure 1:**
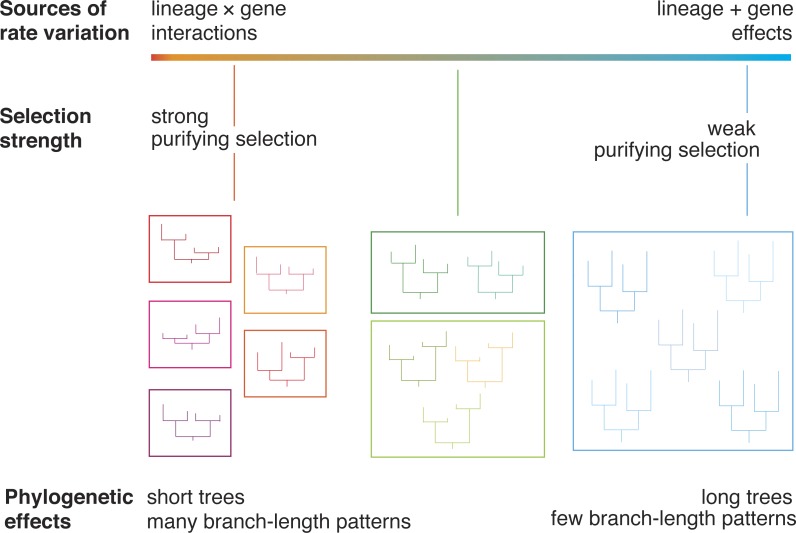
A diagram illustrating the relationship between evolutionary rate and phylogenetic branch-length clusters. Genes that are under strong purifying selection have low rates of evolution, producing short phylogenetic trees. Genes whose evolution is dominated by drift have long phylogenetic trees. We posit that these genes will group into a small number of clusters of branch-length patterns. These genes are primarily subject to lineage effects, which act on a whole-genome scale. In contrast, genes under strong purifying selection experience gene-by-lineage interactions, which lead to distinctive patterns of among-lineage rate variation across genes. These genes will be dispersed into many separate clusters.

Drift and selection tend to have different impacts on evolutionary rates, leading to patterns of rate variation that can be detected using phylogenetic methods (Fig. 1). Furthermore, different genes are subject to varying degrees of selective constraint, leading to measurable disparities in evolutionary rates. For example, functionally important genes tend to evolve slowly because many of the encoded amino acids are under strong selective constraint (Dickerson, 1971). A simple way to detect these “gene effects” is to examine the branch lengths of the gene trees. Genes that are subject to weak selective constraints are expected to yield trees with longer branches, representing a larger total amount of genetic change. In contrast, when genetic change is retarded by purifying selection, genes are expected to yield trees with shorter branches.

The relative impacts of drift and selection also vary across lineages, depending on population size (Ohta, 1992). For example, species with small populations are expected to evolve rapidly because of the dominance of genetic drift (Ohta, 1987). In addition, differences in life-history traits, such as generation time, can produce rate heterogeneity among branches in the tree (Bromham, 2009). Genes that are subject to the same “lineage effects” share the same pattern of relative branch lengths across the tree (Ho & Duchêne, 2014).

Gene and lineage effects can interact to produce “residual effects” (Gillespie, 1991; Muse & Gaut, 1997). Consider two genes, *A* and *B*, sampled from two taxa, *x* and *y*. Both genes are responsible for important biological functions, such that their evolution is constrained. However, gene *A* is under stronger purifying selection in taxon *x* than in taxon *y*. Gene *B* is subject to the reverse conditions, with weaker purifying selection in taxon *x* and stronger selection in taxon *y*. As a consequence, the tree for gene *A* has a longer branch leading to taxon *y* and a shorter branch leading to taxon *x*, whereas the converse is true in the tree for gene *B*. Thus, the trees for these two genes display disparate branch-length patterns.

On a genomic scale, there might be many different patterns of among-lineage rate variation (Ho, 2014; Snir, 2014). These can be identified by statistically clustering gene trees according to their branch-length patterns (Duchêne & Ho, 2015; Duchêne, Foster & Ho, 2016). Each distinct cluster of gene trees identified by this method represents a group of genes that have been subject to a particular combination of gene effects and lineage effects. In the terminology used in previous studies, these genes can be regarded as being governed by the same “pacemaker” (Snir, Wolf & Koonin, 2012; Wolf, Snir & Koonin, 2013). However, this term implies that groups of genes are subject to an underlying evolutionary driving force. Here we simply refer to these groups as clusters of genes that share the same branch-length patterns.

We expect interactions between gene effects and lineage effects to be more common under conditions of selection, because the strength and direction of selection is unlikely to be uniform across species. Therefore, we predict that genes under strong selection will group into many clusters and yield trees with short branches (Fig. 1). In contrast, we predict that genes that are under much weaker selection will group into few branch-rate clusters and yield trees with long branches. Under these conditions, most rate variation is due to lineage effects, such as those caused by differences in generation time. These lineage effects act on a genome-wide scale (Gillespie, 1991), such that different genes share the same pattern of branch-length variation.

In light of the relationships described above, we predict that the clustering of genes according to their branch-length variation is associated with evolutionary rates (Fig. 1). We hypothesize an observable link between evolutionary rate and the dispersion of phylogenetic patterns. This prediction can be tested by analysing genomic data using a phylogenetic approach, because drift and selection leave different signatures in the gene trees. Here we analyse 955 genes from 15 species: two hemimetabolous insects and 13 holometabolous insects. The latter group of insects undergo complete metamorphosis as part of their development. Holometabola arose more than 350 million years ago (Tong et al., 2015) and is extraordinarily diverse: its members include those that are eusocial (Wilson & Hölldobler, 2005), parasites (Libersat, Delago & Gal, 2009), long-distance migrators (Chapman, Reynolds & Wilson, 2015), and ecological engineers (Losey & Vaughan, 2006). They represent a large proportion of the global biomass and are responsible for the bulk of ecological functions on land. We find that as evolutionary rate increases, genes are assigned to fewer branch-length clusters. The results of our analyses point to a general trend that can be tested using genomic data from other groups of organisms.

## Methods

We used maximum likelihood to infer the phylogeny of 15 species of insects (Table S1A). Our data set is based on that analysed by Peters et al. (2014), which originally comprised 1,343 amino acid sequences from 88 species. We filtered this data set in order to remove missing data, producing a subset of 955 amino acid sequences from 15 species (Table S1B). The insects in our analysis are: two bees (*Apis mellifera* and *Bombus terrestris*), two ants (*Linepithema humile* and *Pogonomyrmex barbatus*), a wasp (*Nasonia vitripennis*), three mosquitos (*Anopheles gambiae*, *Aedes aegypti*, and *Culex quinquefasciatus*), three flies (*Drosophila melanogaster*, *Drosophila persimilis*, and *Drosophila sechellia*), a beetle (*Tribolium castaneum*), the silkworm (*Bombyx mori*), a louse (*Pediculus humanus*), and an aphid (*Acyrthosiphon pisum*).

Because we were interested in the relationship between tree length and branch-length patterns, our analyses required the topologies of the gene trees to be mutually congruent. We checked for any substantial differences in topologies between gene trees by clustering them using the *k*-means Partitioning Around Medoids (PAM) algorithm (Kaufman & Rousseeuw, 2009). This method looks for dissimilarity in the data and characterizes variation using representative medoids. In our approach, we considered the pairwise distances between topologies based on the PH85 metric (Penny & Hendy, 1985). We represented these distances in two-dimensional space using multidimensional scaling. As such, each data point corresponds to a gene tree. The algorithm consists of randomly selecting *k* of *n* data points as the medoids. This procedure is repeated until the assignment of the data points to medoids does not change. Because this method does not automatically determine the optimal number of clusters, we calculated a measure of goodness of fit, the Gap statistic (described by Tibshirani, Walther & Hastie, 2001), for a range of values of *k* (1–50), and we selected the *k* with the highest Gap value.

We found strong support for a single cluster of tree topologies, whereby every gene supported the same set of evolutionary relationships among the 15 species of insects. Our topology matches that of previously published insect phylogenies (e.g., Peters et al., 2014; Misof et al., 2014). Accordingly, we inferred the maximum-likelihood tree from a data set comprising the 955 genes in concatenation, using RAxML v8.1 (Stamatakis, 2014). Based on this estimate of the tree topology, we optimized the branch lengths for each gene. Thus, the resulting gene trees shared the same topology but had their own sets of maximum-likelihood branch lengths. The same substitution model, GTR+G with four categories of site rates, was used to estimate the branch lengths for each gene tree. We ran ten replicates of each search and chose the tree with the highest likelihood score.

Using these data, we first tested the assumption that evolutionary rates are associated with the strength of purifying selection. To do this, we determined the relative average rate in each gene by taking the sum of the expected number of substitutions along all of the branches in the corresponding gene tree (i.e., the tree length). We then plotted gene-specific ratios of radical and conservative amino acid substitutions, referred to as the Kr/Kc ratio, against the lengths of the corresponding gene trees. The Kr/Kc ratio is a commonly used and is a robust indicator of selection pressure (Hughes & Nei, 1988; Hanada, Shiu & Li, 2007). It is calculated from protein data, so it is more resistant to the impacts of sequence saturation than Dn/Ds, the equivalent ratio for nucleotide data (Smith & Smith, 1996). Our method of identifying radical and conserved substitutions is similar to that of Zhang (2000). We used a model-free, non-parametric approach to estimate this ratio. This statistic has a similar interpretation to the Kr/Kc ratio (Zhang, 2000), but the absolute values are expected to be different because Kr/Kc is estimated using an explicit substitution model and phylogenetic tree. Although our method is biased towards radical substitutions, with a consequent skew in our results, it provides a fast estimate of the degree of selection. Regardless of the absolute values of Kr/Kc, we consider that our comparison is valid because our data set contains the same set of genes for all taxa, such that they have evolved over the same timescale and are expected to have similar levels of saturation at sites under weak selective constraints. The code for this method is available at www.github.com/kjuntong/tree-length.

We then tested for a relationship between evolutionary rate and the clustering of genes by their branch-length patterns. We assigned genes to clusters by grouping them according to their branch-length patterns using a Gaussian mixture model (GMM) clustering algorithm from the Python machine learning toolkit, Scikit-learn (Pedregosa et al., 2011). GMM algorithms assign data to multivariate normal components and appear to work well when used to identify clusters of branch-length patterns (Duchêne, Foster & Ho, 2016). Importantly, this method clusters the gene trees by their pattern of branch lengths (lineage effects), but not their overall relative evolutionary rate (gene effects). In this study, we did not aim to find the optimal number of clusters for the data; instead, we wished to test our hypothesis using different numbers of clusters. Therefore, we compared the results obtained using different numbers of clusters, from five to 100. We expect that a scheme with few clusters would not provide sufficient resolution to allow us to detect the hypothesized relationship, whereas a scheme with many clusters relative to the total number of genes carries the risk of overfitting. The gene trees were ranked according to evolutionary rate, as denoted by tree length, and divided into deciles. For each tree-length decile, we identified the number of clusters of branch-length patterns that were represented, and plotted these results in a histogram. We then performed a Kendall rank correlation test to measure the association between evolutionary rate and cluster size.

We tested whether our clustering method carried the risk of producing a spurious relationship between tree length and number of clusters of branch-length patterns. To do this, we simulated three sets of 300 gene trees based on the median tree length, the 10th percentile of tree lengths, and the 90th percentile of tree lengths from the insect data. Each tree was generated by simulation according to one of 40 branch-length patterns. Sequence evolution was simulated on each tree to produce an alignment of 353 amino acids, the mean sequence length of the loci in our insect data set. If our method is not biased, we expect that each of the three tree-length categories (short, medium, and long) will contain the same number of branch-length patterns.

In addition to testing the role of evolutionary rate, we investigated whether the clustering of genes by their branch-length patterns could be explained by gene function. Our data set is poorly annotated, which is typical of large data sets generated by high-throughput sequencing. This limited the scope of our investigation to enzymes because enzyme commission (EC) numbers were available for only a subset of our data. EC numbers refer to particular catalytic processes that are enabled by the enzymes. These classifications were available for 297 genes in our data set, but other genes either had incomplete annotations or did not encode enzymes. We looked at the number of clusters of branch-length patterns represented for each of six EC numbers. To correct for an imbalance in the number of genes within each EC category, we divided the number of represented clusters by the number of genes.

Finally, we fitted a random forest classifier to test whether the tree length, ratio of radical and conserved amino acid substitutions, or EC number could predict the cluster assignments of the genes (Liaw & Werner, 2002). Predictive accuracy was quantified using Gini coefficients, a measure of statistical dispersion. A variable with a Gini coefficient of 1 predicts the data perfectly, whereas a coefficient of 0 indicates that the variable is not predictive at all.

## Results

**Figure 2:**
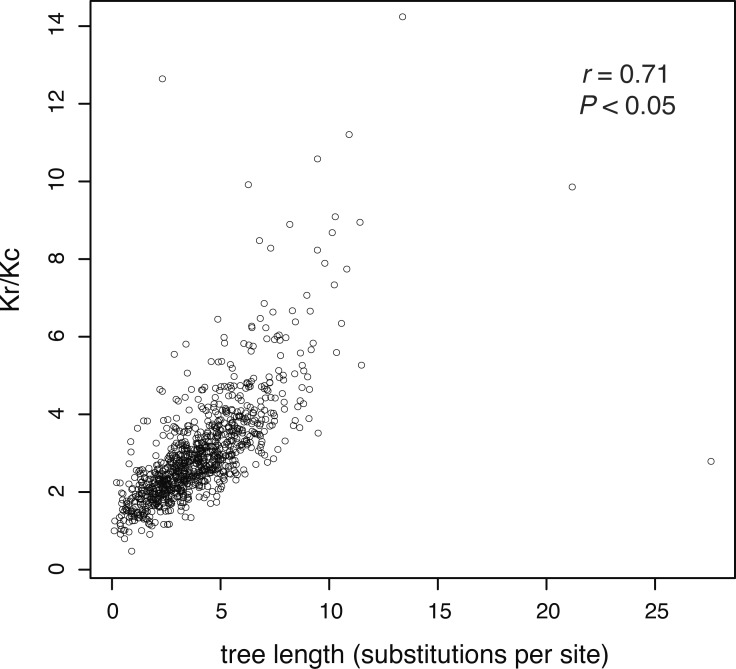
The ratio of estimated radical to non-radical amino acid substitutions (Kr/Kc) shows a positive relationship with evolutionary rate, as measured by gene-tree length. Each point represents one of 955 gene trees. High Kr/Kc values indicate that radical substitutions outnumber non-radical substitutions, reflecting weak selective constraints. Thus, this plot shows that the strength of purifying selection is negatively correlated with evolutionary rate.

In our analysis of 955 amino acid sequences, we first tested the assumption that evolutionary rate is linked to the strength of purifying selection. We found a positive relationship between the ratio of radical and conservative amino acid substitutions and evolutionary rate (as measured by gene-tree length), meaning that more rapidly evolving genes are under weaker purifying selection (Fig. 2).

**Figure 3:**
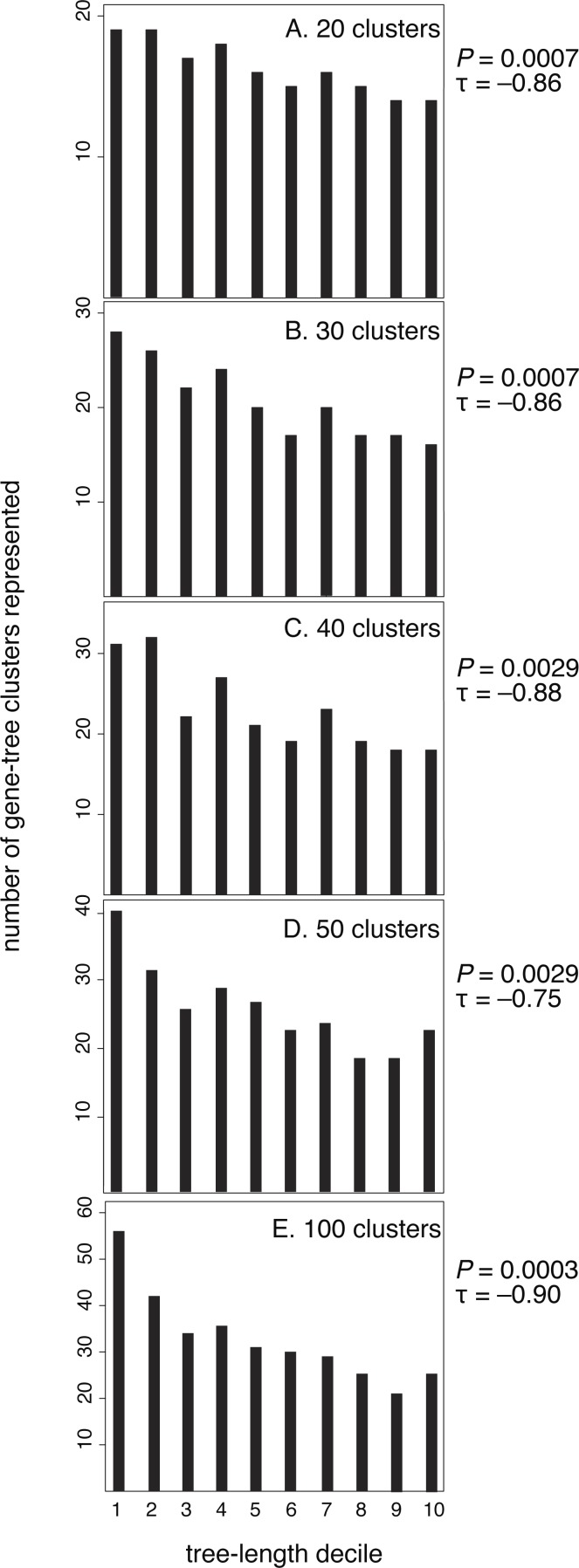
Genes with the longest trees have fewer branch-length patterns than the decile of genes with the shortest trees. Here, each gene has been sorted incrementally into a decile category according to its tree length, where decile 10 contains the longest 10% of trees. Tree length reflects the rate of molecular evolution that has been experienced by a gene, measured in substitutions per site. For each of the five branch-rate clustering schemes (20, 30, 40, 50, and 100 clusters), deciles of genes with higher rates are assigned to fewer clusters.

After confirming the relationship between rate and purifying selection for our data set, we tested our prediction of a relationship between evolutionary rate (tree length) and the clustering of genes by their branch-length patterns (Fig. 3). Our results confirmed this, showing that slowly evolving genes group in many clusters whereas rapidly evolving genes group in fewer clusters. Kendall rank correlations found significant relationships for five clustering schemes: 20 clusters (*P* = 0.0007, *τ* =  − 0.86), 30 clusters (*P* = 0.0007, *τ* =  − 0.86), 40 clusters (*P* = 0.0029, *τ* =  − 0.88), 50 clusters (*P* = 0.0029, *τ* =  − 0.75), and 100 clusters (*P* = 0.0003, *τ* =  − 0.90). We also conducted five- and ten-cluster analyses, but these schemes provided a low level of resolution and we were unable to identify a relationship between the evolutionary rate and clusters of branch-length patterns.

Slowly evolving genes yield short phylogenetic trees that are prone to stochastic estimation errors. Such estimation errors have the potential to confound our analysis by artificially producing variegated patterns of branch lengths. However, it is unlikely that the signal we detect is an artefact because the pattern remains apparent even when we exclude the slowest two deciles of genes. When we excluded the slowest two deciles of genes for each clustering scheme, our results are as follows: 20 clusters (*P* = 0.0078, *τ* =  − 0.79), 30 clusters (*P* = 0.0102, *τ* =  − 0.77), 40 clusters (*P* = 0.0237, *τ* =  − 0.67), 50 clusters (*P* = 0.044, *τ* =  − 0.59), and 100 clusters (*P* = 0.004, *τ* =  − 0.84).

To evaluate the robustness of our clustering method, we repeated our analysis using two additional data sets. We used a 17-taxon data set and a 10-taxon data set that have approximately 250 fewer and more genes than the original data set, respectively. The former consists of 707 genes and the latter 1192 genes, compared with the 955 genes in the original 15-taxon data set. The 17-taxon data set includes the mosquitos *Aedes albopictus* and *Anopheles funestus*, in addition to those represented in the 15-taxon data set (Table S2A). The 10-taxon data set excludes *Drosophila persimilis*, *Drosophila sechellia*, *Bombyx mori*, *Bombus terrestris*, and *Culex quinquefasciatus* from the 15-taxon data set (Table S3A). For both of these data sets, we recovered the same negative relationship between evolutionary rate and the number of clusters of branch-length patterns (Figs. S1A and S1B).

**Figure 4:**
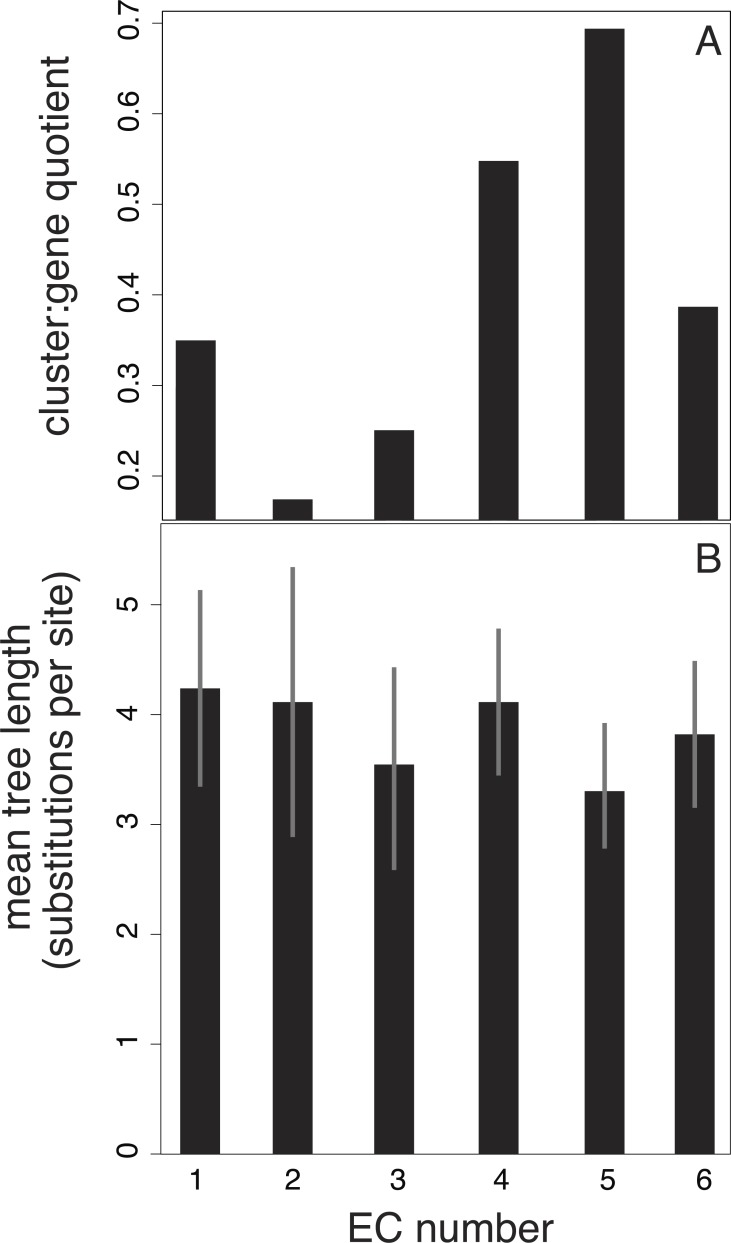
Relationship between EC number and clusters of branch-length patterns for 297 genes in our data set. Each EC number represents a collection of genes that share a common enzyme function. EC number 5, in which genes code for transferases (enzymes that move chemical functional groups from one molecule to another), contains genes with the shortest trees and that are grouped into the largest number of clusters of branch-length patterns. EC number 2 contains genes that are grouped into the smallest number of clusters. EC number 2 codes for isomerases, which are enzymes that convert molecules from one isomer to another. The results are based on a 40-cluster scheme.

We also investigated potential links between branch-length patterns and gene function. We isolated the genes that coded for enzymes in our 15-taxon data set and found that of this subset, isomerase genes (EC number 2) are more likely to group in the same cluster than the genes assigned to other EC numbers (Fig. 4). In contrast, transferase genes (EC number 5) are represented across many clusters.

Lastly, we fitted a random forest classifier to compare the influence of different factors on the assignment of genes to clusters. We found that the length of the gene tree has the best predictive accuracy, with a Gini coefficient of 0.64, for explaining the cluster assignment of a gene. This was followed by our Kr/Kc ratio and the EC number, with respective coefficients of 0.60 and 0.25. However, the classifier has overall low predictive accuracy, suggesting that more gene features might need to be considered to provide a more complete and satisfying model for cluster assignment. This can be improved in the future with further progress in genome annotation. We hope that our results will form the basis for future investigation into the question of whether evolutionary rate is linked to phylogenetic patterns.

## Discussion

### Evolutionary rate informs structure of branch-length patterns

Our analyses reveal that the clustering of branch-length patterns across genes can be at least partly explained by the competing effects of selection and drift. Genes that are the most weakly selected are subject to the vagaries of drift, and they tend to have the highest evolutionary rates across the genome. The main driver of rate heterogeneity in these genes is lineage effects, which explains our finding that large groups of rapidly evolving genes share the same branch-length patterns. The most well studied cause of lineage effects is that of differences in generation time (e.g., Thomas et al., 2010; Weller & Wu, 2015). Generation time has a negative relationship with evolutionary rate because genome replication occurs more infrequently in species with long generations than in those with short generations. This is tempered by the fact that long-lived species tend to have small populations, where drift is the dominant driver of molecular evolution and leads to a higher evolutionary rate (Ohta & Kimura, 1971). However, theoretical examinations suggest that certain mutagenic conditions allow the fixation of neutral mutations to be independent of population size (Welch, Eyre-Walker & Waxman, 2008).

A key problem in our attempt to describe the clustering of branch-length patterns across genes is the effect of fluctuating selection pressures over time. As population sizes change, the effectiveness of selection can increase or decrease. Furthermore, the fitness effects of individual mutations can vary through time, with the potential for selection that is realized under new environmental and ecological conditions (Dykhuizen & Hartl, 1980; Ohta, 1992). The converse might also be true: as selection dynamics shift, the magnitude of selection acting upon a gene might vary over time. Nevertheless, our phylogenetic approach is able to detect an underlying signal through these various sources of noise.

The clustering of branch-length patterns across genes that we observe here reflects groups of genes that share the same temporal patterns of rate variation. The clusters represented in the most rapidly evolving decile of genes might differ from one another by the shifting balance between selection and drift that has occurred over time. For instance, the sets of genes that display two different branch-length patterns might have experienced the same total amount of evolutionary change due to drift, but differ in the periods of time in which they were subject to selection and drift, thereby generating different branch-length patterns. Our results suggest that in the clusters of the most rapidly evolving genes, these sources of fluctuation are genome-wide factors.

Among the most slowly evolving genes, there is a variety of branch-length patterns because of gene-by-lineage interactions that lead to highly heterogeneous evolutionary rates. Genes that have evolved under these conditions are probably important housekeeping genes, such as those that encode histone or ribosomal proteins. These genes would be subject to strong purifying selection.

### Enzyme function and branch-length patterns

The results of our analyses suggest that isomerases are more likely to share the same branch-length pattern compared to other enzymes (Fig. 4). Interestingly, isomerases are more likely to evolve new functions in different EC classes (Martinez Cuesta et al., 2014). Such isomerase sequences might possess latent potential for selection (Dykhuizen & Hartl, 1980), whereby long periods of drift produce a stream of raw genetic variation that can be subject to selection under particular conditions (Ohta, 1987; Ohta, 1992). We speculate that if this is the case, selection is probably occurring at the secondary or tertiary level of protein structure because the trees of the isomerase genes display fewer types of branch-length patterns, indicating that they are subject to little selection pressure at the sequence level. Alternatively, the sharing of branch-length patterns might partly indicate the presence of protein-protein interactions (Lovell & Robertson, 2010).

Our investigation of the relationship between enzyme function and clustering of branch-length patterns is limited in its statistical power. Despite our correction for the imbalance in the number of genes represented across the six EC categories, three of the six categories have 13 or fewer genes; these relatively small groups of genes might have had a large bearing on the results (Fig. 4). Further clouding any signal in the data set is the fact that enzyme function can change without substantial alterations to the nucleotide sequence (Martinez Cuesta et al., 2014). Enzymes can also exhibit ‘promiscuity’, whereby they evolve to catalyse new suites of reactions in addition to their normal functions (O’Brien & Herschlag, 1999; Duarte, Amrein & Kamerlin, 2013). This uncertain correspondence between amino acid changes and biological function, the ultimate target of selection, is potentially a contributor to the statistical noise in our clustering analyses. Amino acid substitutions are also thought to be more insensitive to generation-time effects than nucleotide substitutions, particularly nucleotide changes occurring in non-coding regions, because proteins are more likely to be targets of selection (Ohta, 1992).

### Implications for phylogenomic analysis

Identifying the relationship between evolutionary rates and branch-length patterns across genes provide some useful insights into how genome-scale data might be handled in phylogenetic analysis. There is a need for new analytical methods to extract phylogenetic and temporal signals from genome-scale data without creating excessive computational demands (Kumar & Hedges, 2016; Tong, Lo & Ho, 2016). One promising new approach involves data-clustering to identify subsets of genes that share similar evolutionary characteristics (Duchêne, Molak & Ho, 2014; Mirarab et al., 2014). These techniques have already been used in phylogenomic analyses of mammals (Dos Reis et al., 2012), birds (Jarvis et al., 2014), and insects (Misof et al., 2014).

Our results show that slowly evolving genes tend to yield trees with different patterns of branch lengths. These genes are especially useful for studying ancient divergences because they have experienced less saturation, but they also display greater variation across genes in terms of their among-lineage rate heterogeneity. Therefore, understanding the variation in branch-length patterns across genes has important practical implications for evolutionary dating using molecular clocks (Duchêne & Ho, 2014). Any molecular dating study must be based on a compromise between selecting genes with an appropriate rate of evolution, and selecting genes to minimize the variation in patterns of among-lineage rate heterogeneity.

## Conclusions

In summary, our analysis of insects has revealed that the variation in branch-length patterns across genes can be at least partly explained by the different impacts of drift and selection, which produce predictable patterns of rate variation. This is in spite of the noise created by the complexities of the evolutionary process over hundreds of millions of years. The trends that we report here should be understood as an initial demonstration of phylogenetic tools in studying mutation over a vast timescale. Further detailed annotation of genomes and improved methodologies will open the way for deeper insights into the impacts of gene function on shaping phylogenetic information. We also hope that our results will spur the discovery of other widespread patterns in genome evolution and lead to improvements in phylogenomic analysis.

##  Supplemental Information

10.7717/peerj.3241/supp-1 Figure S1 Additional analyses Analyses of genes from two additional data subsets (of 10 and 17 taxa) confirm that genes with long trees group into fewer clusters of branch-length patterns than do genes with short trees. This relationship is found for five different clustering schemes (20, 30, 40, 50, and 100 clusters). Here, each gene has been sorted incrementally into a decile category according to its tree length, where decile 10 contains the longest 10% of trees. The 10-taxon data set contains 1192 genes and the 17-taxon data set contains 707 genes.

10.7717/peerj.3241/supp-2 Figure S2 Simulation study Results from our simulation study in which we analysed three sets of 300 gene trees. These three sets of trees were based on: (i) the 10th percentile of tree lengths of the 15-taxon data set (short trees); (ii) the median tree length (median trees); or (iii) the 90th percentile of tree lengths (long trees). Error bars represent the range of values over ten replicates. There is no relationship between evolutionary rate and number of branch-length patterns, which is in contrast with the results from our analysis of insect genomic data (Fig. 3).

10.7717/peerj.3241/supp-3 Supplemental Information 1 Supplementary tables

## Additional Information and Declarations

### Data Availability

The following information was supplied regarding data availability: Gihub: www.github.com/kjuntong/tree-length.
