# Supplementary figures and images for: The impacts of drift and selection on genomic evolution in insects
Source: PeerJ. 2017 Apr 27;5:e3241. doi: 10.7717/peerj.3241 (PMC5410144; doi:10.7717/peerj.3241)

10-taxon dataset

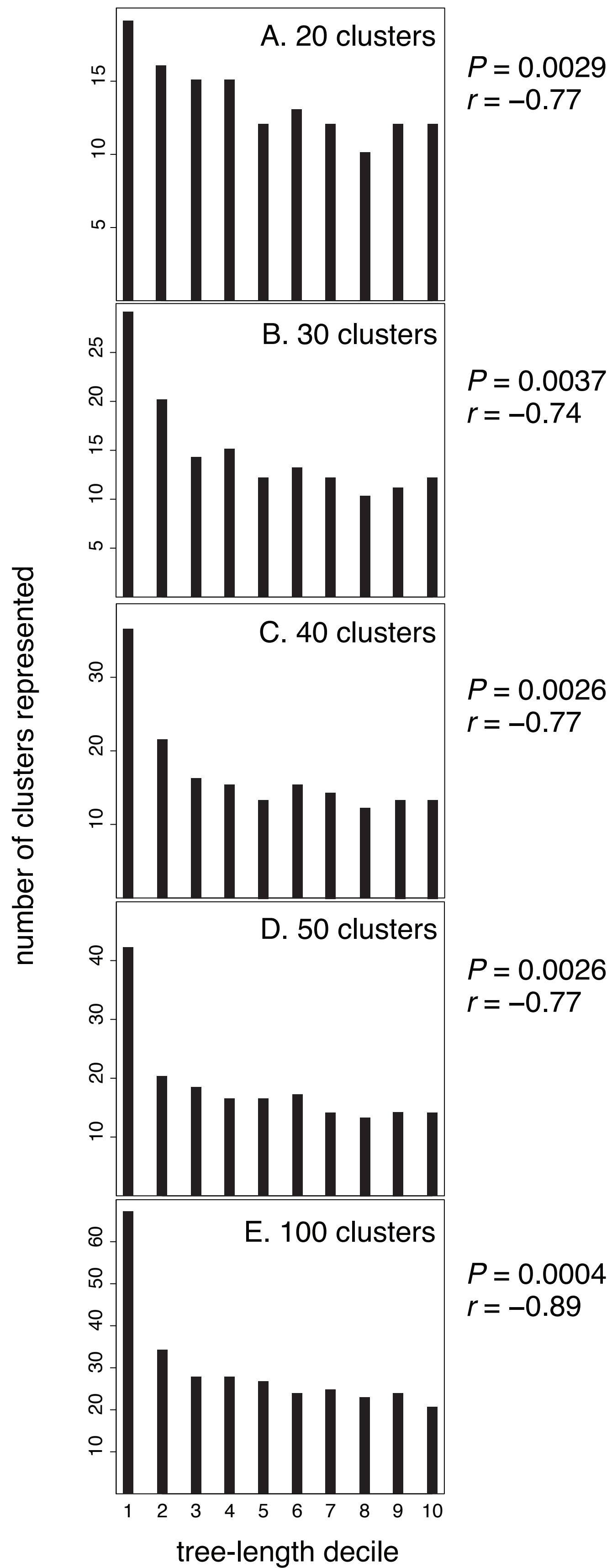

17-taxon dataset

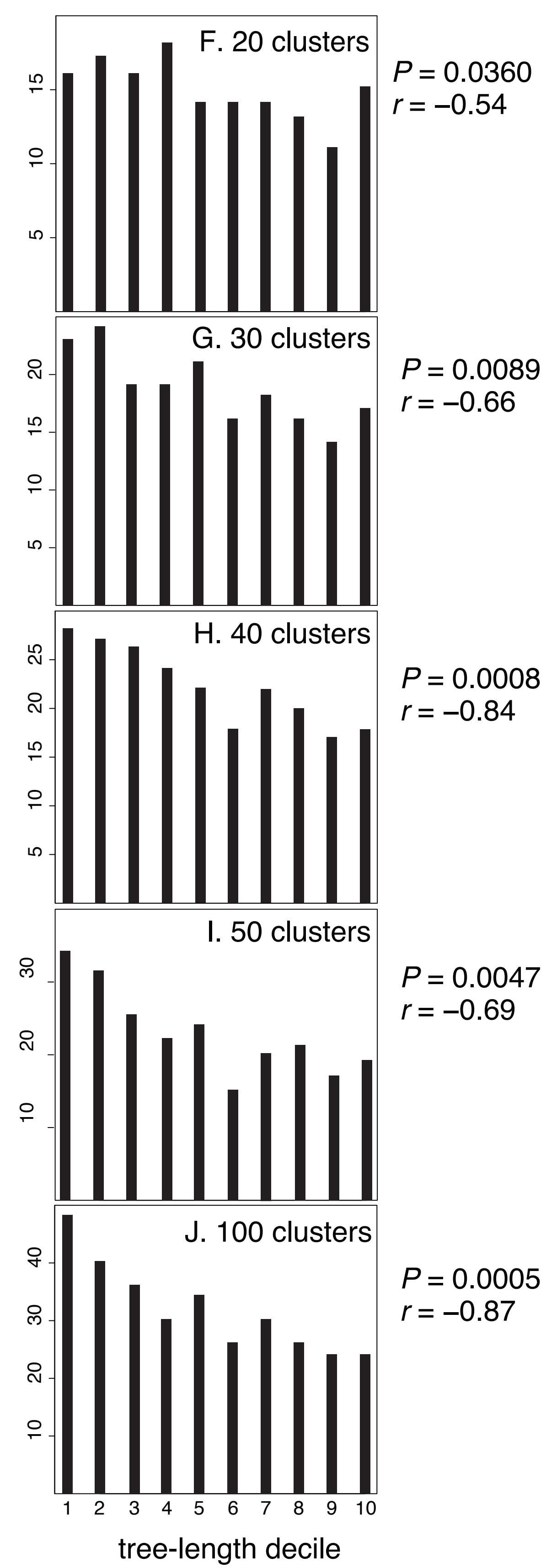

Supplement: Figure S1 — Analyses of genes from two additional data subsets (of 10 and 17 taxa) confirm that genes with long trees group into fewer clusters of branch-length patterns than do genes with short trees. This relationship is found for five different clustering schemes (20, 30, 40, 50, and 100 clusters). Here, each gene has been sorted incrementally into a decile category according to its tree length, where decile 10 contains the longest 10% of trees. The 10-taxon data set contains 1192 genes and the 17-taxon data set contains 707 genes. [file peerj-05-3241-s001.pdf]

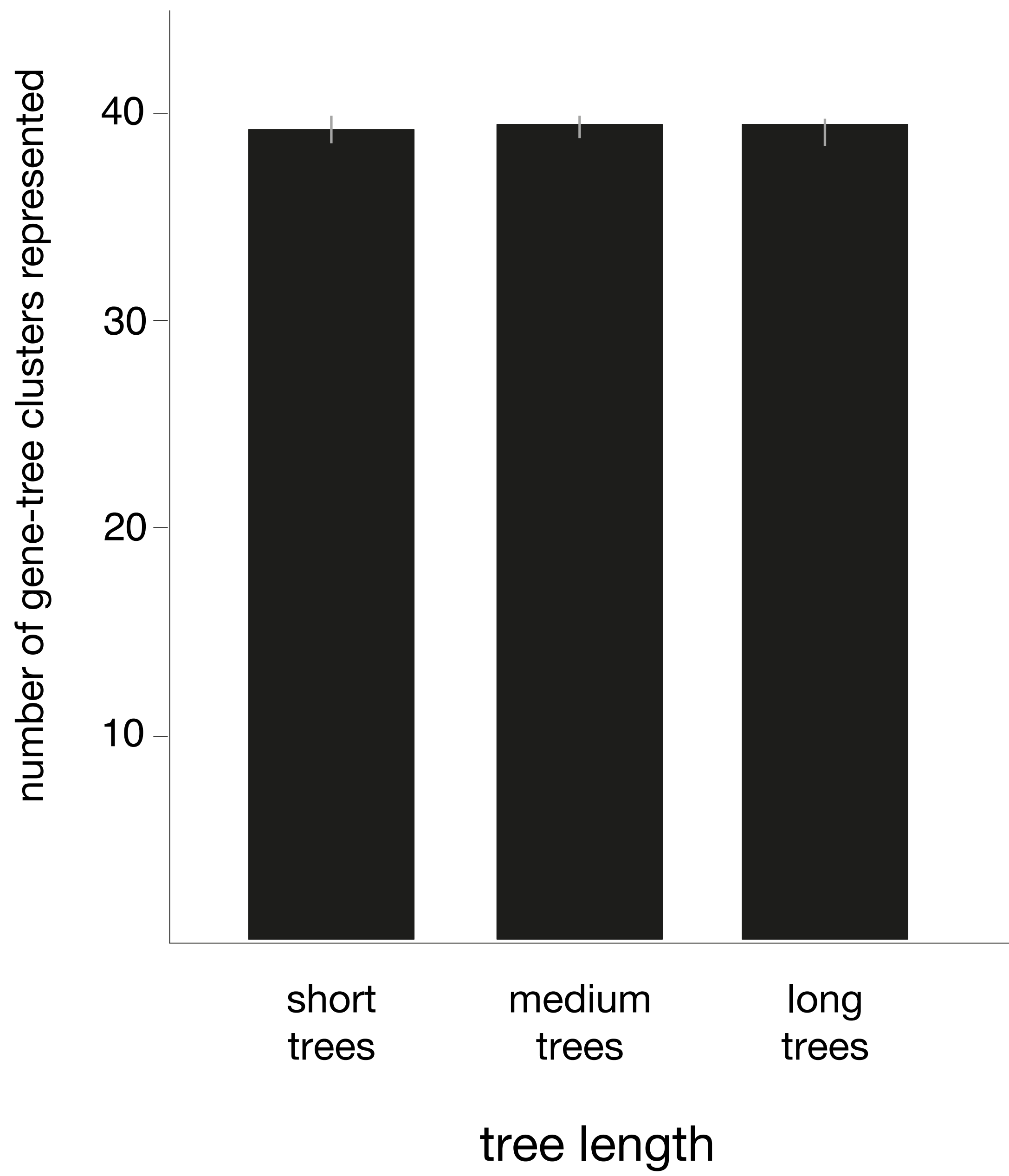

Supplement: Figure S2 — Results from our simulation study in which we analysed three sets of 300 gene trees. These three sets of trees were based on: (i) the 10th percentile of tree lengths of the 15-taxon data set (short trees); (ii) the median tree length (median trees); or (iii) the 90th percentile of tree lengths (long trees). Error bars represent the range of values over ten replicates. There is no relationship between evolutionary rate and number of branch-length patterns, which is in contrast with the results from our analysis of insect genomic data (Fig. 3). [file peerj-05-3241-s002.pdf]
