# Supplementary material for: The impacts of drift and selection on genomic evolution in insects
Source: PeerJ. 2017 Apr 27;5:e3241. doi: 10.7717/peerj.3241 (PMC5410144; doi:10.7717/peerj.3241)
Supplement: Supplemental Information 1 [file peerj-05-3241-s003.pdf]

Supplementary Table S1a. The 15 insect taxa used in our analysis. We used a subset of data from a larger data set retrieved from Peters et al. (2014): https://mynotebook.labarchives.com/share/ubulin/MC4wfDIzNDAzLzAvVHJlZU5vZGUvMjA0NzAzNzkzMHwwLjA. Our subset comprised 15 insects from six separate orders and nine distinct families. Listed here is taxonomic information and the corresponding database in which each organism's sequence data is stored.

Taxonomic group	Order, taxonomic subgroup	Family	Species	Source
Hexapoda, Paraneoptera	Hemiptera, Sternorrhyncha	Aphididae	*Acyrthosiphon pisum*	Aphidbase
Hexapoda, Paraneoptera	Phthiraptera	Pediculidae	*Pediculus humanus*	Aphidbase
Hexapoda, Holometabola	Hymenoptera, Apocrita	Apidae	*Apis mellifera*	Beebase
Hexapoda, Holometabola	Hymenoptera, Apocrita	Apidae	*Bombus terrestris*	NCBI TSA
Hexapoda, Holometabola	Hymenoptera, Apocrita	Pteromalidae	*Nasonia vitripennis*	Hymenopterage nomebase
Hexapoda, Holometabola	Hymenoptera, Apocrita	Formicidae	*Linepithema humile*	Hymenopterage nomebase
Hexapoda, Holometabola	Hymenoptera, Apocrita	Formicidae	*Pogonomyrmex barbatus*	Hymenopterage nomebase
Hexapoda, Holometabola	Coleoptera, Polyphaga	Tenebrionidae	*Tribolium castaneum*	Beetlebase
Hexapoda, Holometabola	Lepidoptera, Ditrysia	Bombycidae	*Bombyx mori*	Silkworm
Hexapoda, Holometabola	Diptera, lower dipterans	Culicidae	*Aedes aegypti*	Vectorbase
Hexapoda, Holometabola	Diptera, lower dipterans	Culicidae	*Anopheles gambiae*	Vectorbase
Hexapoda, Holometabola	Diptera, lower dipterans	Culicidae	*Culex quinquefasciatus*	Vectorbase
Hexapoda, Holometabola	Diptera, Brachycera	Drosophilidae	*Drosophila melanogaster*	Flybase
Hexapoda, Holometabola	Diptera, Brachycera	Drosophilidae	*Drosophila persimilis*	Flybase
Hexapoda, Holometabola	Diptera, Brachycera	Drosophilidae	*Drosophila sechellia*	Flybase

Supplementary Table S1b. A list of the 955 genes in the 15-taxon data set. These sequence names correspond to data from Peters et al. (2014).

Sequence name	Sequence length (amino acids)	Sequence name	Sequence length (amino acids)
EOG4CRJJB	401	EOG47M0GT	668
EOG4N8PQZ	190	EOG4WH75Q	206
EOG4PC8C8	160	EOG4T4BDV	343
EOG495XBQ	234	EOG4DZ0CZ	810
EOG4N2Z7D	301	EOG4BRV4R	397
EOG4H18F0	395	EOG44J13C	533
EOG4NK9G1	123	EOG46HDW4	497
EOG43J9QW	182	EOG4905V7	515
EOG4SJ418	135	EOG4QBZQ6	646
EOG4GMSGZ	296	EOG483BPQ	426
EOG4CC2MR	114	EOG466T5T	203
EOG4TTF3Q	263	EOG4J6Q9J	262
EOG46Q5CN	195	EOG49323P	263
EOG4H9W5J	202	EOG4ZCRQ1	295
EOG4J9KJN	263	EOG4CC2K6	406
EOG498SM6	121	EOG4Q5787	232
EOG43TXFQ	197	EOG4XGXJV	310
EOG4D7WRW	169	EOG48PK4M	419
EOG4N031K	98	EOG4NK9DK	575
EOG4CNPBM	98	EOG4HDRCN	423
EOG434TRW	257	EOG4WSTVK	563
EOG48KPXK	196	EOG4W3R60	705
EOG4BK3Q7	131	EOG49CNSJ	779
EOG4254CQ	211	EOG40P2S2	488
EOG473NB7	201	EOG4CZ90Q	644
EOG4STQQM	194	EOG431ZJP	165
EOG41RNFN	70	EOG4612Q5	228
EOG4WSTVS	435	EOG4Z617N	177

EOG49S4SJ	156	EOG4ZCRQR	168
EOG4S1RT6	258	EOG4D258S	291
EOG4RJDMX	177	EOG495XB5	403
EOG4D258F	305	EOG46WWVF	191
EOG41G1PB	461	EOG4J9KK6	166
EOG43TXF4	334	EOG4M0CN0	270
EOG4PRR8W	401	EOG479CTV	93
EOG483BQK	213	EOG4FN33K	260
EOG4H44PB	319	EOG45QFZM	395
EOG4CJT2K	383	EOG4X9622	725
EOG4P2NN4	205	EOG40VT89	268
EOG41VHP0	183	EOG4CZ91V	229
EOG41NS5B	484	EOG4905VQ	185
EOG480GG7	268	EOG4N2Z85	145
EOG4SN074	342	EOG4V6X1Q	577
EOG46HDW0	368	EOG4254BM	1002
EOG4G1K2F	188	EOG4TB2WN	300
EOG4QNKG1	260	EOG4PNW24	417
EOG4HMGW0	256	EOG4H70X6	406
EOG44B8MT	325	EOG4TMPNB	144
EOG4GTHZZ	193	EOG469PDM	244
EOG43R26B	193	EOG4Z08QV	411
EOG415F06	211	EOG47H486	418
EOG4NGF6C	226	EOG43TXDR	429
EOG4M0CN6	152	EOG40K6J9	308
EOG408KV1	181	EOG48GTP9	199
EOG434TRS	188	EOG4254BT	505
EOG4D257W	515	EOG45DV8W	102
EOG4CNP9X	218	EOG49GJ1T	342
EOG43J9Q5	355	EOG4Q83GM	440
EOG4C86BR	371	EOG4K0P66	864
EOG476HJM	392	EOG4CC2K5	746

EOG4ZGMZ8	212	EOG4DNCP9	717
EOG4BG7FJ	321	EOG4RFJD3	127
EOG4ZS7NF	284	EOG4P5HW4	217
EOG4QV9Z1	241	EOG40RZ16	351
EOG4FFBN0	255	EOG483BPH	672
EOG45X6FJ	362	EOG4V9S8J	823
EOG43R26K	382	EOG4WWQ3D	626
EOG4FTTM2	96	EOG4RR52B	859
EOG451C9W	221	EOG4GHX78	485
EOG49KD9K	188	EOG4CFXTT	429
EOG40P2T4	120	EOG4N02ZS	822
EOG45X6G3	235	EOG4JSXQF	888
EOG4CZ928	116	EOG47H481	760
EOG4XD2BD	178	EOG4FTTJK	594
EOG4CZ920	165	EOG49322M	1146
EOG4S7H8T	247	EOG4GB5R1	809
EOG4FQZBQ	372	EOG4VQ872	1092
EOG4WM3F2	123	EOG4W9GNM	1631
EOG40CG32	221	EOG4X3FKP	459
EOG402VC8	193	EOG4NP5NG	542
EOG437PZZ	552	EOG4GF205	519
EOG4K0P6P	288	EOG4CRJJC	247
EOG480GGM	208	EOG4ZW3W4	535
EOG4X963M	123	EOG408KST	836
EOG44QRKH	363	EOG4280N0	119
EOG402VC2	219	EOG4Q2C0S	408
EOG4NCJZC	234	EOG4JWSZD	644
EOG4C2FW6	297	EOG4V41SP	467
EOG4BCC6Q	265	EOG4FXPSK	524
EOG4NCJZ9	313	EOG41896R	523
EOG4DFN74	306	EOG4VT4G6	532
EOG4KWHC1	533	EOG4GXD5P	1114

EOG4WWQ3W	391	EOG45DV78	738
EOG480GFR	433	EOG4K0P69	484
EOG4GB5S4	240	EOG4XWDHS	214
EOG43J9QS	200	EOG4DZ0DP	853
EOG4G79HN	452	EOG4ZGMX8	657
EOG4254C2	361	EOG4QJQ6C	531
EOG4JSXRC	219	EOG4NGF5D	1444
EOG4N5TH6	222	EOG4KPRVW	588
EOG4V6X21	442	EOG40P2RK	980
EOG4S4N1F	394	EOG42Z386	774
EOG42FR32	321	EOG43TXD9	1189
EOG49KD8H	705	EOG495X9P	698
EOG4QNKF1	567	EOG4G79H4	1131
EOG4XWDHQ	202	EOG4HQC34	746
EOG4SF7S7	114	EOG4PVMHD	907
EOG49CNT7	302	EOG4RFJBC	764
EOG44J13Z	272	EOG4S1RS2	815
EOG4PK0V8	195	EOG4ZS7N2	687
EOG4F4QVV	929	EOG4WWQ4H	204
EOG4NVX56	245	EOG47WM82	126
EOG4N8PPZ	358	EOG4FFBN3	108
EOG45QG01	398	EOG4280M6	314
EOG4QNKG0	279	EOG4J6Q92	543
EOG4KSN43	400	EOG4DV46Q	123
EOG40ZPGM	646	EOG4K6DPQ	438
EOG4SN08D	101	EOG4K6DPW	340
EOG46DJNN	209	EOG4612PN	246
EOG4J3V2T	245	EOG431ZH6	355
EOG4NP5PB	229	EOG42NGJW	347
EOG4RR53D	189	EOG4JM695	163
EOG4MW6RD	392	EOG4P8D3Q	265
EOG4N8PR5	147	EOG451CB2	123

EOG4KWHCN	212	EOG4BVQDV	160
EOG48GTP6	349	EOG4CFXV8	251
EOG4T1G64	185	EOG4FFBM8	301
EOG4FJ6VS	199	EOG4ZS7NW	302
EOG42BVVN	216	EOG41ZCW6	351
EOG41C5GC	215	EOG4W3R70	205
EOG4K3JFV	353	EOG4Z617W	219
EOG4D7WR0	395	EOG4K6DQP	100
EOG4D51J1	128	EOG49KD8X	376
EOG4K6DPN	423	EOG4M90B3	294
EOG4THTD9	231	EOG4W0VZ3	684
EOG4FTTKP	377	EOG415DZX	303
EOG4ZGMXV	377	EOG45B012	379
EOG4BK3QD	118	EOG4CVDTD	192
EOG4H44NZ	410	EOG444J4V	314
EOG4S7H8Z	227	EOG4573S3	325
EOG4ZS7N6	493	EOG4254C9	343
EOG41897G	228	EOG41NS5G	321
EOG4V15M3	130	EOG48PK4D	473
EOG48KPWS	329	EOG45QFZH	386
EOG437Q17	149	EOG4G4F8J	431
EOG479CSS	282	EOG4DNCPR	359
EOG4NZSD1	246	EOG4BRV4Q	471
EOG47PVRM	251	EOG4XGXHZ	896
EOG4K3JH1	62	EOG4KWHCQ	516
EOG479CT3	185	EOG4QJQ70	334
EOG4KPRWD	394	EOG49CNV2	151
EOG4VHHRG	487	EOG4MGQT7	211
EOG4MKM1B	481	EOG4XPP12	461
EOG46WWV7	254	EOG4Q5782	425
EOG42NGK6	314	EOG4GF204	630
EOG4WDBX1	421	EOG47D80W	1036

EOG4J0ZVF	132	EOG43XSNX	339
EOG4866ZS	178	EOG4CJT28	663
EOG4JM68T	224	EOG4GTHXT	463
EOG4KWHBW	476	EOG41JWXX	622
EOG4WSTWC	255	EOG4PVMHF	834
EOG4GHX8F	200	EOG4FQZBS	188
EOG4M909H	645	EOG4XD29W	273
EOG4RV19S	283	EOG4FBGCG	347
EOG40ZPH0	364	EOG46HDW6	356
EOG4CZ92H	78	EOG43R25Z	421
EOG4X69TT	327	EOG4PK0T2	491
EOG44XH1T	610	EOG4RFJCT	156
EOG4C5B3F	553	EOG4MSBJK	159
EOG4V6X36	117	EOG4TB2WP	393
EOG4ZPCF3	291	EOG47H48C	325
EOG437Q0H	291	EOG422854	123
EOG46T1M3	295	EOG4BCC6M	251
EOG4P8D41	151	EOG4N2Z7K	372
EOG48GTP3	228	EOG4C86C0	249
EOG4FN333	395	EOG4F1VN8	384
EOG4J0ZV8	199	EOG4X962F	297
EOG434TRG	322	EOG4JHB1N	171
EOG4Q83H2	288	EOG48GTNQ	397
EOG4PC8BD	338	EOG4S7H81	647
EOG40GB9P	245	EOG4PC8B4	566
EOG408KT0	649	EOG40K6HV	624
EOG4V15M1	205	EOG4PC8B0	760
EOG49GJ2K	237	EOG4PZGRB	1375
EOG4CJT2Q	314	EOG4KWHBQ	635
EOG44XH2Q	257	EOG42JMB5	424
EOG4VT4GW	262	EOG4QJQ67	694
EOG47H487	333	EOG480GG8	262

EOG45B00H	342	EOG4T4BDH	937
EOG4JQ2GV	360	EOG40P2T6	121
EOG444J5J	197	EOG4M909S	353
EOG4DJHG2	348	EOG45MKQW	404
EOG40CG3J	94	EOG4QFTZK	389
EOG4DNCQC	207	EOG4D7WQG	745
EOG4RJDMC	221	EOG4MGQS6	773
EOG4T4BDW	385	EOG4W0VXW	1023
EOG4CJT23	321	EOG4Q2C0B	1337
EOG4PVMHP	191	EOG43TXDC	1094
EOG46DJNF	240	EOG4GB5QX	1183
EOG40ZPH3	299	EOG4MPG7Z	850
EOG48GTNR	403	EOG4QBZQB	791
EOG434TR7	454	EOG4ZW3VW	982
EOG4NVX4R	289	EOG441NWW	292
EOG480GGX	162	EOG4ZW3WQ	284
EOG4FXPTJ	184	EOG40P2RR	563
EOG42FR3M	181	EOG4BCC67	625
EOG489365	210	EOG4SF7R6	521
EOG4RJDMF	242	EOG4WPZMR	420
EOG4X3FKN	363	EOG4N5TG2	499
EOG4S7H8D	358	EOG4CFXTD	394
EOG4PRR9M	193	EOG4G4F91	333
EOG48SFCC	614	EOG49CNSG	909
EOG4T4BDK	397	EOG49S4RG	417
EOG4PK0TN	204	EOG4B5MQF	441
EOG4SN06Z	327	EOG43XSNM	554
EOG451C9K	307	EOG4KD54S	832
EOG48PK4G	337	EOG4STQPH	786
EOG476HJR	373	EOG49P8H5	1140
EOG4H44PZ	147	EOG4X0KBT	655
EOG4FJ6VQ	203	EOG42Z38F	437

EOG4HHMN7	292	EOG4WH74X	566
EOG470S29	522	EOG43TXD8	1223
EOG4JM68W	187	EOG45X6DT	1636
EOG42NGKB	252	EOG48PK40	938
EOG44F4VX	300	EOG498SJP	838
EOG47M0J4	204	EOG49ZW74	929
EOG4FBGCM	311	EOG49ZW7B	592
EOG4QBZQJ	399	EOG4B2RG5	644
EOG4T1G6B	259	EOG4XGXHT	1531
EOG4C86BS	407	EOG4WWQ4X	128
EOG4X3FKR	381	EOG4S1RTC	166
EOG4S4N1Q	274	EOG4Q83HR	153
EOG408KV3	208	EOG4K6DPG	426
EOG4PVMJK	195	EOG4WWQ51	119
EOG4SF7S1	340	EOG408KVF	177
EOG4V15K0	753	EOG4BG7G0	160
EOG49S4SD	180	EOG4B5MR2	267
EOG41896F	529	EOG45B00V	304
EOG4S4N17	193	EOG405QM9	190
EOG4PVMJ2	330	EOG47PVS4	240
EOG476HK3	285	EOG42BVV5	309
EOG4GQNQ1	658	EOG4B5MR1	292
EOG43J9QT	188	EOG4SXKZV	165
EOG46Q5BW	384	EOG4K3JG5	247
EOG4NGF6V	172	EOG4V41T1	346
EOG4KKWP7	183	EOG42RBTD	156
EOG48CZDG	457	EOG41VHP3	169
EOG4TB2WK	208	EOG4DFN6W	419
EOG4BVQCQ	666	EOG4RBP48	340
EOG431ZHM	395	EOG44J13R	256
EOG4QFV0H	146	EOG4Q5791	83
EOG45HQGM	472	EOG4ZKH62	281

EOG4RN8W3	123	EOG4ZGMXK	442
EOG4STQPQ	516	EOG4FQZBH	239
EOG4280KK	574	EOG4D51GV	392
EOG4MGQT0	401	EOG4J3V3D	92
EOG441NXR	134	EOG46M94F	242
EOG4B2RH7	186	EOG4JSXQT	417
EOG4S7H8F	405	EOG4RN8V4	227
EOG4GB5RZ	253	EOG4ZW3WB	473
EOG466T5W	240	EOG48GTNW	332
EOG415F0F	214	EOG41JWXV	340
EOG447DCV	287	EOG48W9KZ	377
EOG4G4F87	750	EOG4QBZQN	327
EOG4VQ87G	421	EOG4W0VZX	276
EOG4Z616H	631	EOG4MSBJ0	301
EOG4WWQ3Z	340	EOG46Q5BR	308
EOG4H44NH	642	EOG4PK0SS	688
EOG4WPZNB	402	EOG49CNSP	618
EOG48SFCM	314	EOG4N5TH5	123
EOG4FXPSJ	483	EOG4THTCM	287
EOG4BCC5W	657	EOG4612PB	397
EOG422849	301	EOG4C86CB	180
EOG4GHX7C	430	EOG4JHB1Z	128
EOG4M37V5	498	EOG44F4W2	205
EOG4254BR	495	EOG4B8GZ9	372
EOG4HX3KF	339	EOG4QNKF8	656
EOG4QFTZR	317	EOG4FTTJJ	619
EOG473N9J	414	EOG45X6FQ	533
EOG4SQVFH	613	EOG41JWXG	348
EOG42283X	470	EOG4JQ2GH	555
EOG44QRJT	704	EOG4NVX5Q	138
EOG4SQVGB	251	EOG4GQNQQ	309
EOG4Z6174	394	EOG4NP5P9	228

EOG49KD8C	787	EOG4GQNR6	196
EOG41ZCVW	492	EOG4DV45T	348
EOG4C5B3K	530	EOG400048	133
EOG4NZSCZ	326	EOG4Q5784	266
EOG48CZFJ	402	EOG4FXPT9	264
EOG44MW9Z	572	EOG40ZPH8	270
EOG498SM1	133	EOG4GHX81	180
EOG41ZCXD	125	EOG476HK8	193
EOG4GF21C	185	EOG4ZCRQC	270
EOG47WM7S	199	EOG4PRR95	361
EOG49P8JX	132	EOG49S4RJ	420
EOG4547JK	249	EOG4GTHZ9	310
EOG48PK4Z	216	EOG4V6X31	147
EOG41JWZ3	241	EOG4KWHC9	364
EOG4GQNQJ	305	EOG4BRV5B	353
EOG479CT2	265	EOG4N2Z6T	922
EOG4SQVH1	124	EOG4QV9XS	198
EOG4HX3M8	213	EOG4MSBJ1	235
EOG4DJHGW	115	EOG4QZ66C	131
EOG4C2FWV	132	EOG489371	120
EOG4VHHSR	108	EOG42V714	726
EOG49W11S	113	EOG4H9W5F	253
EOG473NB1	185	EOG4SQVFV	397
EOG4SXKZ2	297	EOG4GHX70	716
EOG4FXPTT	142	EOG4X3FKF	513
EOG4GB5SM	107	EOG43TXDM	582
EOG46HDX9	122	EOG43J9PK	744
EOG4PVMJ8	294	EOG49322Q	911
EOG4Z08R1	669	EOG45B00C	903
EOG486707	75	EOG48SFCB	467
EOG48SFD8	150	EOG476HJB	552
EOG44QRM3	189	EOG49ZW85	650

EOG4MGQT1	307	EOG4KH1DV	455
EOG4GHX7Q	298	EOG444J47	666
EOG4N5THC	139	EOG4GTHXS	725
EOG44MW9V	673	EOG40P2RN	885
EOG4MW6RQ	261	EOG43J9PG	1102
EOG41897C	201	EOG46DJMM	945
EOG4SN089	106	EOG4DR7X7	820
EOG4XD2BC	146	EOG4KKWNJ	743
EOG48CZF9	221	EOG48KPWG	330
EOG4H18FV	207	EOG4H9W5N	196
EOG4NS1XK	179	EOG4WDBXF	290
EOG4MPG8P	313	EOG4NS1XS	173
EOG49P8HT	383	EOG43TXF5	369
EOG4STQR3	120	EOG4VQ87R	358
EOG4F1VP6	223	EOG47SQZW	515
EOG4Q2C13	329	EOG4GQNQ9	392
EOG4V6X2Q	184	EOG4ZW3VZ	799
EOG495XB1	404	EOG4H18DN	799
EOG4BZKMJ	630	EOG4HX3MK	190
EOG44XH2N	356	EOG463XX2	711
EOG45DV8C	278	EOG4MW6RP	255
EOG48PK5H	142	EOG42BVTH	751
EOG431ZJJ	106	EOG40VT7F	852
EOG447DDC	172	EOG4WDBWX	526
EOG4HQC4F	211	EOG44F4VT	299
EOG4BRV57	194	EOG4RFJBN	565
EOG4D51HB	308	EOG4FTTKH	447
EOG4R4XPD	90	EOG4C5B3Z	344
EOG42Z39F	154	EOG44B8M6	730
EOG479CS5	381	EOG4SQVFR	755
EOG4GTHZ4	360	EOG4PK0ST	431
EOG4H18F7	347	EOG4JWSZP	479

EOG4QFTZV	210	EOG4866XH	660
EOG4BG7DZ	578	EOG47WM6K	748
EOG476HK6	292	EOG46T1KH	640
EOG4DFN7R	170	EOG47D81K	524
EOG44J13J	497	EOG42JM9D	567
EOG4RBP47	410	EOG42FR34	673
EOG4K6DQF	141	EOG437PZV	757
EOG4GB5RK	309	EOG49S4R4	1635
EOG4FN33M	173	EOG4DV456	698
EOG4S1RTG	177	EOG4G1K15	1080
EOG45HQHB	206	EOG4J9KHW	593
EOG4W0VZH	433	EOG4PRR8H	1293
EOG4KWHD5	144	EOG4R22CK	1585
EOG49KD96	411	EOG4RJDK9	1094
EOG47SR0R	197	EOG4RN8TB	747
EOG4F1VN3	676	EOG4RV197	862
EOG4HMGWP	153	EOG4SJ3ZN	1358
EOG4GTJ0C	112	EOG4V15JR	1051
EOG4K3JGS	110	EOG4WM3CP	877
EOG44XH27	357	EOG46T1KW	338
EOG4H70WN	580	EOG4T76ND	447
EOG4F4QW2	502	EOG47H48J	305
EOG4BK3NV	605	EOG4DBS08	360
EOG4ZKH5D	442	EOG4H9W4P	394
EOG47WM6Z	676	EOG44B8MK	410
EOG4MSBHJ	340	EOG46HDVH	862
EOG480GFH	733	EOG43BK87	133
EOG4MGQSB	595	EOG42RBS6	662
EOG4ZGMXD	508	EOG4TQJW1	361
EOG47SQZM	734	EOG4C86BG	553
EOG49W10Q	362	EOG4V9S93	351
EOG4SQVFN	801	EOG49S4R9	730

EOG41ZCVS	936	EOG4RR52Q	397
EOG4D2584	433	EOG470S2X	323
EOG4WDBWQ	808	EOG48PK5R	76
EOG43N5XG	931	EOG45QFZQ	288
EOG4S4N0W	1167	EOG4HT7C9	185
EOG4QNKFZ	440	EOG402VB4	539
EOG4TDZ4B	438	EOG4TMPMD	446
EOG44J137	380	EOG4QFV08	198
EOG470S3P	109	EOG4RFJCF	325
EOG4FBGCB	361	EOG4G79HH	502
EOG48GTNP	383	EOG4612PM	403
EOG4JDFS4	430	EOG476HJ9	488
EOG45MKQ6	672	EOG4MSBH9	514
EOG4D7WQP	571	EOG4RBP4W	203
EOG4DFN6P	490	EOG4BRV54	387
EOG4P8D2Z	712	EOG48935H	571
EOG4V9S8M	564	EOG4RXWJS	505
EOG4V15JZ	433	EOG48SFC2	607
EOG42JM9M	530	EOG44QRKQ	295
EOG4MPG8H	378	EOG4C2FVQ	500
EOG415DZC	568	EOG45QFZ2	933
EOG476HJ7	532	EOG476HHX	1354
EOG4GB5R8	637	EOG4BVQCF	1118
EOG415DZ5	1075	EOG4SN075	339
EOG4FQZ9G	943	EOG4SN07Z	214
EOG4ZS7N0	1006	EOG4NS1W7	1566
EOG4X963K	131	EOG4JWT04	507
EOG4FXPV6	91	EOG4GF1ZX	703
EOG4JWT03	289	EOG405QJW	1036
EOG408KTH	359	EOG40ZPGC	1528
EOG4GXD6X	180	EOG42BVV0	402
EOG4CNPB6	255	EOG476HJ4	676

EOG473NB2	241	EOG4HQC39	721
EOG4HQC4T	112	EOG42FR30	320
EOG4X69VH	191	EOG495X9T	546
EOG466T5H	441	EOG45TB5Z	756
EOG476HK9	207	EOG47SQZF	2245
EOG4F1VP2	216	EOG4BNZWN	919
EOG4866Z2	307	EOG4DFN6C	1062
EOG4T4BF8	284	EOG4FJ6TJ	966
EOG4N8PQJ	337	EOG4HMGW1	588
EOG4M90B0	335	EOG4K6DP0	1236
EOG48KPXZ	202	EOG4N02ZQ	1128
EOG4PK0V1	122	EOG4QV9WQ	1204
EOG4TTF2V	661	EOG4RN8T9	859
EOG40ZPH5	371	EOG4X0KB4	1211
EOG48W9MZ	192	EOG44MWBP	309
EOG45QFZ6	604	EOG42284D	293
EOG4TX9BB	314	EOG49P8HR	423
EOG44F4VM	450	EOG4S1RSB	437
EOG444J4D	548	EOG45DV7S	496
EOG451C92	409	EOG4547HN	661
EOG4FXPSS	352	EOG4RR53N	170
EOG42Z399	225	EOG4NCJZ7	307
EOG4MSBHD	415	EOG4QFTZT	325
EOG4KD552	722	EOG4P2NM8	631
EOG4GHX7X	306	EOG4JM67H	813
EOG44MWB6	466	EOG4MW6S9	129
EOG4B2RHM	126	EOG4WM3CX	358
EOG4N5TGH	288	EOG4M0CKD	2085
EOG44MWC8	202	EOG41RND4	469
EOG4N8PQK	293	EOG47M0GX	583
EOG4GQNQV	224	EOG4RFJBQ	592
EOG4F4QX0	338	EOG45MKQ5	612

EOG4HT7CC	169	EOG4C2FVD	688
EOG473N9V	321	EOG4XKSSV	280
EOG4DBS0V	184	EOG4C2FW3	302
EOG4905VS	211	EOG4NS1XD	267
EOG447DC9	443	EOG408KSV	855
EOG4GHX74	501	EOG4F7M3R	685
EOG40ZPHF	321	EOG4BNZWP	547
EOG4V6X2J	201	EOG4DJHF5	2092
EOG4BG7GM	133	EOG4TMPKT	986
EOG4JDFT3	211	EOG41ZCX0	287
EOG4FJ6TS	599	EOG46DJMZ	357
EOG4TX9B3	422	EOG45HQG6	798
EOG4MGQSF	462	EOG4QJQ76	153
EOG4254CR	238	EOG49GJ1H	781
EOG47PVRQ	216	EOG4CRJHR	2692
EOG49KD8K	372	EOG4P2NN5	359
EOG46Q5BV	349	EOG4TB2W2	1132
EOG4PZGS6	229	EOG4MGQSM	435
EOG400038	682	EOG48CZD2	1231
EOG4NK9FZ	132	EOG4HMGVB	579
EOG45X6F3	608	EOG4612NS	939
EOG4JDFSJ	252	EOG4BNZWJ	991
EOG4JSXRB	407	EOG4DFN69	1045
EOG4G4F92	233	EOG4FBGBM	1633
EOG4866XS	452	EOG4G79H3	1319
EOG4MKM1R	388	EOG4GMSFR	1079
EOG473NBF	169	EOG4H18DG	2056
EOG43R25S	498	EOG4JM67D	1052
EOG4KSN49	300	EOG4MCVJ4	1001
EOG40GBB2	239	EOG4MKM10	1605
EOG4J9KHT	642	EOG4MW6QW	984
EOG42NGJJ	481	EOG4TX99J	1834

EOG4BCC5V	816	EOG4WPZNW	352
EOG45B00G	578		

Supplementary Table S2a. A subset of 17 insect taxa. This set of taxa consists of those found in Supplementary Table S1, with the addition of two mosquitos: *Aedes albopictus* and *Anopheles funestus*. We used sequences from these taxa to complement our results from the primary 15-taxon data set.

**Taxonomic group**	**Order, taxonomic subgroup**	**Family**	**Species**	**Source**
Hexapoda, Paraneoptera	Hemiptera, Sternorrhyncha	Aphididae	*Acyrthosiphon pisum*	Aphidbase
Hexapoda, Paraneoptera	Phthiraptera	Pediculidae	*Pediculus humanus*	Aphidbase
Hexapoda, Holometabola	Hymenoptera, Apocrita	Apidae	*Apis mellifera*	Beebase
Hexapoda, Holometabola	Hymenoptera, Apocrita	Apidae	*Bombus terrestris*	NCBI TSA
Hexapoda, Holometabola	Hymenoptera, Apocrita	Pteromalidae	*Nasonia vitripennis*	Hymenopteragenome base
Hexapoda, Holometabola	Hymenoptera, Apocrita	Formicidae	*Linepithema humile*	Hymenopteragenome base
Hexapoda, Holometabola	Hymenoptera, Apocrita	Formicidae	*Pogonomyrmex barbatus*	Hymenopteragenome base
Hexapoda, Holometabola	Coleoptera, Polyphaga	Tenebrionidae	*Tribolium castaneum*	Beetlebase
Hexapoda, Holometabola	Lepidoptera, Ditrysia	Bombycidae	*Bombyx mori*	Silkworm
Hexapoda, Holometabola	Diptera, lower dipterans	Culicidae	*Aedes aegypti*	Vectorbase
Hexapoda, Holometabola	Diptera, lower dipterans	Culicidae	*Anopheles gambiae*	Vectorbase
Hexapoda, Holometabola	Diptera, lower dipterans	Culicidae	*Culex quinquefasciatus*	Vectorbase
Hexapoda, Holometabola	Diptera, lower dipterans	Culicidae	*Aedes albopictus*	NCBI TSA
Hexapoda, Holometabola	Diptera, lower dipterans	Culicidae	*Anopheles funestus*	NCBI TSA
Hexapoda, Holometabola	Diptera, Brachycera	Drosophilidae	*Drosophila melanogaster*	Flybase
Hexapoda, Holometabola	Diptera, Brachycera	Drosophilidae	*Drosophila persimilis*	Flybase
Hexapoda, Holometabola	Diptera, Brachycera	Drosophilidae	*Drosophila sechellia*	Flybase

Supplementary Table S2b. A list of the 707 genes in the 17-taxon data set. These sequence names correspond to data from Peters et al. (2014).

Sequence name	Sequence length (amino acids)	Sequence name	Sequence length (amino acids)
EOG4CRJJB	401	EOG4K6DQF	141
EOG4N8PQZ	190	EOG4GB5RK	309
EOG4PC8C8	160	EOG4FN33M	173
EOG495XBQ	234	EOG4S1RTG	177
EOG4N2Z7D	301	EOG45HQHB	206
EOG4H18F0	395	EOG4W0VZH	433
EOG4NK9G1	123	EOG4KWHD5	144
EOG43J9QW	182	EOG49KD96	411
EOG4SJ418	135	EOG47SR0R	197
EOG4GMSGZ	296	EOG4F1VN3	676
EOG4CC2MR	114	EOG4HMGWP	153
EOG4TTF3Q	263	EOG4GTJ0C	112
EOG46Q5CN	195	EOG4K3JGS	110
EOG4H9W5J	202	EOG44XH27	357
EOG4J9KJN	263	EOG4H70WN	580
EOG498SM6	121	EOG4F4QW2	502
EOG43TXFQ	197	EOG4BK3NV	605
EOG4D7WRW	169	EOG4ZKH5D	442
EOG4N031K	98	EOG47WM6Z	676
EOG4CNPBM	98	EOG4MSBHJ	340
EOG434TRW	257	EOG480GFH	733
EOG48KPXK	196	EOG4MGQSB	595
EOG4BK3Q7	131	EOG4ZGMXD	508
EOG4254CQ	211	EOG47SQZM	734
EOG473NB7	201	EOG49W10Q	362
EOG4STQQM	194	EOG4SQVFN	801
EOG41RNFN	70	EOG41ZCVS	936
EOG4WSTVS	435	EOG4D2584	433
EOG49S4SJ	156	EOG4WDBWQ	808

EOG4S1RT6	258	EOG43N5XG	931
EOG4RJDMX	177	EOG4S4N0W	1167
EOG4D258F	305	EOG4QNKFZ	440
EOG41G1PB	461	EOG4TDZ4B	438
EOG43TXF4	334	EOG44J137	380
EOG4PRR8W	401	EOG470S3P	109
EOG483BQK	213	EOG4FBGCB	361
EOG4H44PB	319	EOG48GTNP	383
EOG4CJT2K	383	EOG4JDFS4	430
EOG4P2NN4	205	EOG45MKQ6	672
EOG41VHP0	183	EOG4D7WQP	571
EOG41NS5B	484	EOG4DFN6P	490
EOG480GG7	268	EOG4P8D2Z	712
EOG4SN074	342	EOG4V9S8M	564
EOG46HDW0	368	EOG4V15JZ	433
EOG4G1K2F	188	EOG42JM9M	530
EOG4QNKG1	260	EOG4MPG8H	378
EOG4HMGW0	256	EOG415DZC	568
EOG44B8MT	325	EOG476HJ7	532
EOG4GTHZZ	193	EOG4GB5R8	637
EOG43R26B	193	EOG415DZ5	1075
EOG415F06	211	EOG4FQZ9G	943
EOG4NGF6C	226	EOG4ZS7N0	1006
EOG4M0CN6	152	EOG4X963K	131
EOG408KV1	181	EOG4FXPV6	91
EOG434TRS	188	EOG4JWT03	289
EOG4D257W	515	EOG408KTH	359
EOG4CNP9X	218	EOG4GXD6X	180
EOG43J9Q5	355	EOG4CNPB6	255
EOG4C86BR	371	EOG473NB2	241
EOG476HJM	392	EOG4HQC4T	112
EOG4ZGMZ8	212	EOG4X69VH	191
EOG4BG7FJ	321	EOG466T5H	441

EOG4ZS7NF	284	EOG476HK9	207
EOG4QV9Z1	241	EOG4F1VP2	216
EOG4FFBN0	255	EOG4866Z2	307
EOG45X6FJ	362	EOG4T4BF8	284
EOG43R26K	382	EOG4N8PQJ	337
EOG4FTTM2	96	EOG4M90B0	335
EOG451C9W	221	EOG48KPXZ	202
EOG49KD9K	188	EOG4PK0V1	122
EOG40P2T4	120	EOG4TTF2V	661
EOG45X6G3	235	EOG40ZPH5	371
EOG4CZ928	116	EOG48W9MZ	192
EOG4XD2BD	178	EOG45QFZ6	604
EOG4CZ920	165	EOG4TX9BB	314
EOG4S7H8T	247	EOG44F4VM	450
EOG4FQZBQ	372	EOG444J4D	548
EOG4WM3F2	123	EOG451C92	409
EOG40CG32	221	EOG4FXPSS	352
EOG402VC8	193	EOG42Z399	225
EOG437PZZ	552	EOG4MSBHD	415
EOG4K0P6P	288	EOG4KD552	722
EOG480GGM	208	EOG4GHX7X	306
EOG4X963M	123	EOG44MWB6	466
EOG44QRKH	363	EOG4B2RHM	126
EOG402VC2	219	EOG4N5TGH	288
EOG4NCJZC	234	EOG44MWC8	202
EOG4C2FW6	297	EOG4N8PQK	293
EOG4BCC6Q	265	EOG4GQNQV	224
EOG4NCJZ9	313	EOG4F4QX0	338
EOG4DFN74	306	EOG4HT7CC	169
EOG4KWHC1	533	EOG473N9V	321
EOG4WWQ3W	391	EOG4DBS0V	184
EOG480GFR	433	EOG4905VS	211
EOG4GB5S4	240	EOG447DC9	443

EOG43J9QS	200	EOG4GHX74	501
EOG4G79HN	452	EOG40ZPHF	321
EOG4254C2	361	EOG4V6X2J	201
EOG4JSXRC	219	EOG4BG7GM	133
EOG4N5TH6	222	EOG4JDFT3	211
EOG4V6X21	442	EOG4FJ6TS	599
EOG4S4N1F	394	EOG4TX9B3	422
EOG42FR32	321	EOG4MGQSF	462
EOG49KD8H	705	EOG4254CR	238
EOG4QNKF1	567	EOG47PVRQ	216
EOG4XWDHQ	202	EOG49KD8K	372
EOG4SF7S7	114	EOG46Q5BV	349
EOG49CNT7	302	EOG4PZGS6	229
EOG44J13Z	272	EOG400038	682
EOG4PK0V8	195	EOG4NK9FZ	132
EOG4F4QVV	929	EOG45X6F3	608
EOG4NVX56	245	EOG4JDFSJ	252
EOG4N8PPZ	358	EOG4JSXRB	407
EOG45QG01	398	EOG4G4F92	233
EOG4QNKG0	279	EOG4866XS	452
EOG4KSN43	400	EOG4MKM1R	388
EOG40ZPGM	646	EOG473NBF	169
EOG4SN08D	101	EOG43R25S	498
EOG46DJNN	209	EOG4KSN49	300
EOG4J3V2T	245	EOG40GBB2	239
EOG4NP5PB	229	EOG4J9KHT	642
EOG4RR53D	189	EOG42NGJJ	481
EOG4MW6RD	392	EOG4BCC5V	816
EOG4N8PR5	147	EOG45B00G	578
EOG4KWHCN	212	EOG47M0GT	668
EOG48GTP6	349	EOG4WH75Q	206
EOG4T1G64	185	EOG4T4BDV	343
EOG4FJ6VS	199	EOG4DZ0CZ	810

EOG42BVVN	216	EOG4BRV4R	397
EOG41C5GC	215	EOG44J13C	533
EOG4K3JFV	353	EOG46HDW4	497
EOG4D7WR0	395	EOG4905V7	515
EOG4D51J1	128	EOG4QBZQ6	646
EOG4K6DPN	423	EOG483BPQ	426
EOG4THTD9	231	EOG466T5T	203
EOG4FTTKP	377	EOG4J6Q9J	262
EOG4ZGMXV	377	EOG49323P	263
EOG4BK3QD	118	EOG4ZCRQ1	295
EOG4H44NZ	410	EOG4CC2K6	406
EOG4S7H8Z	227	EOG4Q5787	232
EOG4ZS7N6	493	EOG4XGXJV	310
EOG41897G	228	EOG48PK4M	419
EOG4V15M3	130	EOG4NK9DK	575
EOG48KPWS	329	EOG4HDRCN	423
EOG437Q17	149	EOG4WSTVK	563
EOG479CSS	282	EOG4W3R60	705
EOG4NZSD1	246	EOG49CNSJ	779
EOG47PVRM	251	EOG40P2S2	488
EOG4K3JH1	62	EOG4CZ90Q	644
EOG479CT3	185	EOG431ZJP	165
EOG4KPRWD	394	EOG4612Q5	228
EOG4VHHRG	487	EOG4Z617N	177
EOG4MKM1B	481	EOG4ZCRQR	168
EOG46WWV7	254	EOG4D258S	291
EOG42NGK6	314	EOG495XB5	403
EOG4WDBX1	421	EOG46WWVF	191
EOG4J0ZVF	132	EOG4J9KK6	166
EOG4866ZS	178	EOG4M0CN0	270
EOG4JM68T	224	EOG479CTV	93
EOG4KWHBW	476	EOG4FN33K	260
EOG4WSTWC	255	EOG45QFZM	395

EOG4GHX8F	200	EOG4X9622	725
EOG4M909H	645	EOG40VT89	268
EOG4RV19S	283	EOG4CZ91V	229
EOG40ZPH0	364	EOG4905VQ	185
EOG4CZ92H	78	EOG4N2Z85	145
EOG4X69TT	327	EOG4V6X1Q	577
EOG44XH1T	610	EOG4254BM	1002
EOG4C5B3F	553	EOG4TB2WN	300
EOG4V6X36	117	EOG4PNW24	417
EOG4ZPCF3	291	EOG4H70X6	406
EOG437Q0H	291	EOG4TMPNB	144
EOG46T1M3	295	EOG469PDM	244
EOG4P8D41	151	EOG4Z08QV	411
EOG48GTP3	228	EOG47H486	418
EOG4FN333	395	EOG43TXDR	429
EOG4J0ZV8	199	EOG40K6J9	308
EOG434TRG	322	EOG48GTP9	199
EOG4Q83H2	288	EOG4254BT	505
EOG4PC8BD	338	EOG45DV8W	102
EOG40GB9P	245	EOG49GJ1T	342
EOG408KT0	649	EOG4Q83GM	440
EOG4V15M1	205	EOG4K0P66	864
EOG49GJ2K	237	EOG4CC2K5	746
EOG4CJT2Q	314	EOG4DNCP9	717
EOG44XH2Q	257	EOG4RFJD3	127
EOG4VT4GW	262	EOG4P5HW4	217
EOG47H487	333	EOG40RZ16	351
EOG45B00H	342	EOG483BPH	672
EOG4JQ2GV	360	EOG4V9S8J	823
EOG444J5J	197	EOG4WWQ3D	626
EOG4DJHG2	348	EOG4RR52B	859
EOG40CG3J	94	EOG4GHX78	485
EOG4DNCQC	207	EOG4CFXTT	429

EOG4RJDMC	221	EOG4N02ZS	822
EOG4T4BDW	385	EOG4JSXQF	888
EOG4CJT23	321	EOG47H481	760
EOG4PVMHP	191	EOG4FTTJK	594
EOG46DJNF	240	EOG49322M	1146
EOG40ZPH3	299	EOG4GB5R1	809
EOG48GTNR	403	EOG4VQ872	1092
EOG434TR7	454	EOG4W9GNM	1631
EOG4NVX4R	289	EOG4X3FKP	459
EOG480GGX	162	EOG4NP5NG	542
EOG4FXPTJ	184	EOG4GF205	519
EOG42FR3M	181	EOG4CRJJC	247
EOG489365	210	EOG4ZW3W4	535
EOG4RJDMF	242	EOG408KST	836
EOG4X3FKN	363	EOG4280N0	119
EOG4S7H8D	358	EOG4Q2C0S	408
EOG4PRR9M	193	EOG4JWSZD	644
EOG48SFCC	614	EOG4V41SP	467
EOG4T4BDK	397	EOG4FXPSK	524
EOG4PK0TN	204	EOG41896R	523
EOG4SN06Z	327	EOG4VT4G6	532
EOG451C9K	307	EOG4GXD5P	1114
EOG48PK4G	337	EOG45DV78	738
EOG476HJR	373	EOG4K0P69	484
EOG4H44PZ	147	EOG4XWDHS	214
EOG4FJ6VQ	203	EOG4DZ0DP	853
EOG4HHMN7	292	EOG4ZGMX8	657
EOG470S29	522	EOG4QJQ6C	531
EOG4JM68W	187	EOG4NGF5D	1444
EOG42NGKB	252	EOG4KPRVW	588
EOG44F4VX	300	EOG40P2RK	980
EOG47M0J4	204	EOG42Z386	774
EOG4FBGCM	311	EOG43TXD9	1189

EOG4QBZQJ	399	EOG495X9P	698
EOG4T1G6B	259	EOG4G79H4	1131
EOG4C86BS	407	EOG4HQC34	746
EOG4X3FKR	381	EOG4PVMHD	907
EOG4S4N1Q	274	EOG4RFJBC	764
EOG408KV3	208	EOG4S1RS2	815
EOG4PVMJK	195	EOG4ZS7N2	687
EOG4SF7S1	340	EOG4WWQ4H	204
EOG4V15K0	753	EOG47WM82	126
EOG49S4SD	180	EOG4FFBN3	108
EOG41896F	529	EOG4280M6	314
EOG4S4N17	193	EOG4J6Q92	543
EOG4PVMJ2	330	EOG4DV46Q	123
EOG476HK3	285	EOG4K6DPQ	438
EOG4GQNQ1	658	EOG4K6DPW	340
EOG43J9QT	188	EOG4612PN	246
EOG46Q5BW	384	EOG431ZH6	355
EOG4NGF6V	172	EOG42NGJW	347
EOG4KKWP7	183	EOG4JM695	163
EOG48CZDG	457	EOG4P8D3Q	265
EOG4TB2WK	208	EOG451CB2	123
EOG4BVQCQ	666	EOG4BVQDV	160
EOG431ZHM	395	EOG4CFXV8	251
EOG4QFV0H	146	EOG4FFBM8	301
EOG45HQGM	472	EOG4ZS7NW	302
EOG4RN8W3	123	EOG41ZCW6	351
EOG4STQPQ	516	EOG4W3R70	205
EOG4280KK	574	EOG4Z617W	219
EOG4MGQT0	401	EOG4K6DQP	100
EOG441NXR	134	EOG49KD8X	376
EOG4B2RH7	186	EOG4M90B3	294
EOG4S7H8F	405	EOG4W0VZ3	684
EOG4GB5RZ	253	EOG415DZX	303

EOG466T5W	240	EOG45B012	379
EOG415F0F	214	EOG4CVDTD	192
EOG447DCV	287	EOG444J4V	314
EOG4G4F87	750	EOG4573S3	325
EOG4VQ87G	421	EOG4254C9	343
EOG4Z616H	631	EOG41NS5G	321
EOG4WWQ3Z	340	EOG48PK4D	473
EOG4H44NH	642	EOG45QFZH	386
EOG4WPZNB	402	EOG4G4F8J	431
EOG48SFCM	314	EOG4DNCPR	359
EOG4FXPSJ	483	EOG4BRV4Q	471
EOG4BCC5W	657	EOG4XGXHZ	896
EOG422849	301	EOG4KWHCQ	516
EOG4GHX7C	430	EOG4QJQ70	334
EOG4M37V5	498	EOG49CNV2	151
EOG4254BR	495	EOG4MGQT7	211
EOG4HX3KF	339	EOG4XPP12	461
EOG4QFTZR	317	EOG4Q5782	425
EOG473N9J	414	EOG4GF204	630
EOG4SQVFH	613	EOG47D80W	1036
EOG42283X	470	EOG43XSNX	339
EOG44QRJT	704	EOG4CJT28	663
EOG4SQVGB	251	EOG4GTHXT	463
EOG4Z6174	394	EOG41JWXX	622
EOG49KD8C	787	EOG4PVMHF	834
EOG41ZCVW	492	EOG4FQZBS	188
EOG4C5B3K	530	EOG4XD29W	273
EOG4NZSCZ	326	EOG4FBGCG	347
EOG48CZFJ	402	EOG46HDW6	356
EOG44MW9Z	572	EOG43R25Z	421
EOG498SM1	133	EOG4PK0T2	491
EOG41ZCXD	125	EOG4RFJCT	156
EOG4GF21C	185	EOG4MSBJK	159

EOG47WM7S	199	EOG4TB2WP	393
EOG49P8JX	132	EOG47H48C	325
EOG4547JK	249	EOG422854	123
EOG48PK4Z	216	EOG4BCC6M	251
EOG41JWZ3	241	EOG4N2Z7K	372
EOG4GQNQJ	305	EOG4C86C0	249
EOG479CT2	265	EOG4F1VN8	384
EOG4SQVH1	124	EOG4X962F	297
EOG4HX3M8	213	EOG4JHB1N	171
EOG4DJHGW	115	EOG48GTNQ	397
EOG4C2FWV	132	EOG4S7H81	647
EOG4VHHSR	108	EOG4PC8B4	566
EOG49W11S	113	EOG40K6HV	624
EOG473NB1	185	EOG4PC8B0	760
EOG4SXKZ2	297	EOG4PZGRB	1375
EOG4FXPTT	142	EOG4KWHBQ	635
EOG4GB5SM	107	EOG42JMB5	424
EOG46HDX9	122	EOG4QJQ67	694
EOG4PVMJ8	294	EOG480GG8	262
EOG4Z08R1	669	EOG4T4BDH	937
EOG486707	75	EOG40P2T6	121
EOG48SFD8	150	EOG4M909S	353
EOG44QRM3	189	EOG45MKQW	404
EOG4MGQT1	307	EOG4QFTZK	389
EOG4GHX7Q	298	EOG4D7WQG	745
EOG4N5THC	139	EOG4MGQS6	773
EOG44MW9V	673	EOG4W0VXW	1023
EOG4MW6RQ	261	EOG4Q2C0B	1337
EOG41897C	201	EOG43TXDC	1094
EOG4SN089	106	EOG4GB5QX	1183
EOG4XD2BC	146	EOG4MPG7Z	850
EOG48CZF9	221	EOG4QBZQB	791
EOG4H18FV	207	EOG4ZW3VW	982

EOG4NS1XK	179	EOG441NWW	292
EOG4MPG8P	313	EOG4ZW3WQ	284
EOG49P8HT	383	EOG40P2RR	563
EOG4STQR3	120	EOG4BCC67	625
EOG4F1VP6	223	EOG4SF7R6	521
EOG4Q2C13	329	EOG4WPZMR	420
EOG4V6X2Q	184	EOG4N5TG2	499
EOG495XB1	404	EOG4CFXTD	394
EOG4BZKMJ	630	EOG4G4F91	333
EOG44XH2N	356	EOG49CNSG	909
EOG45DV8C	278	EOG49S4RG	417
EOG48PK5H	142	EOG4B5MQF	441
EOG431ZJJ	106	EOG43XSNM	554
EOG447DDC	172	EOG4KD54S	832
EOG4HQC4F	211	EOG4STQPH	786
EOG4BRV57	194	EOG49P8H5	1140
EOG4D51HB	308	EOG4X0KBT	655
EOG4R4XPD	90	EOG42Z38F	437
EOG42Z39F	154	EOG4WH74X	566
EOG479CS5	381	EOG43TXD8	1223
EOG4GTHZ4	360	EOG45X6DT	1636
EOG4H18F7	347	EOG48PK40	938
EOG4QFTZV	210	EOG498SJP	838
EOG4BG7DZ	578	EOG49ZW74	929
EOG476HK6	292	EOG49ZW7B	592
EOG4DFN7R	170	EOG4B2RG5	644
EOG44J13J	497	EOG4XGXHT	1531
EOG4RBP47	410		

Supplementary Table S3a. A subset of 10 insect taxa. This is a subset of taxa from Supplementary Table S1. We used sequences from these taxa to complement our results from the primary 15-taxon data set.

**Taxonomic group**	**Order, taxonomic subgroup**	**Family**	**Species**	**Source**
Hexapoda, Paraneoptera	Hemiptera, Sternorrhyncha	Aphididae	*Acyrthosiphon pisum*	Aphidbase
Hexapoda, Paraneoptera	Phthiraptera	Pediculidae	*Pediculus humanus*	Aphidbase
Hexapoda, Holometabola	Hymenoptera, Apocrita	Apidae	*Apis mellifera*	Beebase
Hexapoda, Holometabola	Hymenoptera, Apocrita	Pteromalidae	*Nasonia vitripennis*	Hymenopteragenomebase
Hexapoda, Holometabola	Hymenoptera, Apocrita	Formicidae	*Linepithema humile*	Hymenopteragenomebase
Hexapoda, Holometabola	Hymenoptera, Apocrita	Formicidae	*Pogonomyrmex barbatus*	Hymenopteragenomebase
Hexapoda, Holometabola	Coleoptera, Polyphaga	Tenebrionidae	*Tribolium castaneum*	Beetlebase
Hexapoda, Holometabola	Diptera, lower dipterans	Culicidae	*Aedes aegypti*	Vectorbase
Hexapoda, Holometabola	Diptera, lower dipterans	Culicidae	*Anopheles gambiae*	Vectorbase
Hexapoda, Holometabola	Diptera, Brachycera	Drosophilidae	*Drosophila melanogaster*	Flybase

Supplementary Table S3b. A list of the 1192 genes corresponding to the 10-taxon data set. These sequence names correspond to data from Peters et al. (2014).

Sequence name	Sequence length (amino acids)	Sequence name	Sequence length (amino acids)
EOG4CRJJB	401	EOG4612PN	246
EOG4N8PQZ	190	EOG431ZH6	355
EOG4PC8C8	160	EOG42NGJW	347
EOG495XBQ	234	EOG4JM695	163
EOG4N2Z7D	301	EOG4P8D3Q	265
EOG4H18F0	395	EOG451CB2	123
EOG4NK9G1	123	EOG4BVQDV	160
EOG43J9QW	182	EOG4CFXV8	251
EOG4SJ418	135	EOG4FFBM8	301
EOG4GMSGZ	296	EOG4ZS7NW	302
EOG4CC2MR	114	EOG41ZCW6	351
EOG4TTF3Q	263	EOG4W3R70	205
EOG46Q5CN	195	EOG4Z617W	219
EOG4H9W5J	202	EOG4K6DQP	100
EOG4J9KJN	263	EOG49KD8X	376
EOG498SM6	121	EOG4M90B3	294
EOG43TXFQ	197	EOG4W0VZ3	684
EOG4D7WRW	169	EOG415DZX	303
EOG4N031K	98	EOG45B012	379
EOG4CNPBM	98	EOG4CVDTD	192
EOG434TRW	257	EOG444J4V	314
EOG48KPXK	196	EOG4573S3	325
EOG4BK3Q7	131	EOG4254C9	343
EOG4254CQ	211	EOG41NS5G	321
EOG473NB7	201	EOG48PK4D	473
EOG4STQQM	194	EOG45QFZH	386
EOG41RNFN	70	EOG4G4F8J	431
EOG4WSTVS	435	EOG4DNCPR	359
EOG49S4SJ	156	EOG4BRV4Q	471

EOG4S1RT6	258	EOG4XGXHZ	896
EOG4RJDMX	177	EOG4KWHCQ	516
EOG4D258F	305	EOG4QJQ70	334
EOG41G1PB	461	EOG49CNV2	151
EOG43TXF4	334	EOG4MGQT7	211
EOG4PRR8W	401	EOG4XPP12	461
EOG483BQK	213	EOG4Q5782	425
EOG4H44PB	319	EOG4GF204	630
EOG4CJT2K	383	EOG47D80W	1036
EOG4P2NN4	205	EOG43XSNX	339
EOG41VHP0	183	EOG4CJT28	663
EOG41NS5B	484	EOG4GTHXT	463
EOG480GG7	268	EOG41JWXX	622
EOG4SN074	342	EOG4PVMHF	834
EOG46HDW0	368	EOG4FQZBS	188
EOG4G1K2F	188	EOG4XD29W	273
EOG4QNKG1	260	EOG4FBGCG	347
EOG4HMGW0	256	EOG46HDW6	356
EOG44B8MT	325	EOG43R25Z	421
EOG4GTHZZ	193	EOG4PK0T2	491
EOG43R26B	193	EOG4RFJCT	156
EOG415F06	211	EOG4MSBJK	159
EOG4NGF6C	226	EOG4TB2WP	393
EOG4M0CN6	152	EOG47H48C	325
EOG408KV1	181	EOG422854	123
EOG434TRS	188	EOG4BCC6M	251
EOG4D257W	515	EOG4N2Z7K	372
EOG4CNP9X	218	EOG4C86C0	249
EOG43J9Q5	355	EOG4F1VN8	384
EOG4C86BR	371	EOG4X962F	297
EOG476HJM	392	EOG4JHB1N	171
EOG4ZGMZ8	212	EOG48GTNQ	397
EOG4BG7FJ	321	EOG4S7H81	647

EOG4ZS7NF	284	EOG4PC8B4	566
EOG4QV9Z1	241	EOG40K6HV	624
EOG4FFBN0	255	EOG4PC8B0	760
EOG45X6FJ	362	EOG4PZGRB	1375
EOG43R26K	382	EOG4KWHBQ	635
EOG4FTTM2	96	EOG42JMB5	424
EOG451C9W	221	EOG4QJQ67	694
EOG49KD9K	188	EOG480GG8	262
EOG40P2T4	120	EOG4T4BDH	937
EOG45X6G3	235	EOG40P2T6	121
EOG4CZ928	116	EOG4M909S	353
EOG4XD2BD	178	EOG45MKQW	404
EOG4CZ920	165	EOG4QFTZK	389
EOG4S7H8T	247	EOG4D7WQG	745
EOG4FQZBQ	372	EOG4MGQS6	773
EOG4WM3F2	123	EOG4W0VXW	1023
EOG40CG32	221	EOG4Q2C0B	1337
EOG402VC8	193	EOG43TXDC	1094
EOG437PZZ	552	EOG4GB5QX	1183
EOG4K0P6P	288	EOG4MPG7Z	850
EOG480GGM	208	EOG4QBZQB	791
EOG4X963M	123	EOG4ZW3VW	982
EOG44QRKH	363	EOG441NWW	292
EOG402VC2	219	EOG4ZW3WQ	284
EOG4NCJZC	234	EOG40P2RR	563
EOG4C2FW6	297	EOG4BCC67	625
EOG4BCC6Q	265	EOG4SF7R6	521
EOG4NCJZ9	313	EOG4WPZMR	420
EOG4DFN74	306	EOG4N5TG2	499
EOG4KWHC1	533	EOG4CFXTD	394
EOG4WWQ3W	391	EOG4G4F91	333
EOG480GFR	433	EOG49CNSG	909
EOG4GB5S4	240	EOG49S4RG	417

EOG43J9QS	200	EOG4B5MQF	441
EOG4G79HN	452	EOG43XSNM	554
EOG4254C2	361	EOG4KD54S	832
EOG4JSXRC	219	EOG4STQPH	786
EOG4N5TH6	222	EOG49P8H5	1140
EOG4V6X21	442	EOG4X0KBT	655
EOG4S4N1F	394	EOG42Z38F	437
EOG42FR32	321	EOG4WH74X	566
EOG49KD8H	705	EOG43TXD8	1223
EOG4QNKF1	567	EOG45X6DT	1636
EOG4XWDHQ	202	EOG48PK40	938
EOG4SF7S7	114	EOG498SJP	838
EOG49CNT7	302	EOG49ZW74	929
EOG44J13Z	272	EOG49ZW7B	592
EOG4PK0V8	195	EOG4B2RG5	644
EOG4F4QVV	929	EOG4XGXHT	1531
EOG4NVX56	245	EOG4WWQ4X	128
EOG4N8PPZ	358	EOG4S1RTC	166
EOG45QG01	398	EOG4Q83HR	153
EOG4QNKG0	279	EOG4K6DPG	426
EOG4KSN43	400	EOG4WWQ51	119
EOG40ZPGM	646	EOG408KVF	177
EOG4SN08D	101	EOG4BG7G0	160
EOG46DJNN	209	EOG4B5MR2	267
EOG4J3V2T	245	EOG45B00V	304
EOG4NP5PB	229	EOG405QM9	190
EOG4RR53D	189	EOG47PVS4	240
EOG4MW6RD	392	EOG42BVV5	309
EOG4N8PR5	147	EOG4B5MR1	292
EOG4KWHCN	212	EOG4SXKZV	165
EOG48GTP6	349	EOG4K3JG5	247
EOG4T1G64	185	EOG4V41T1	346
EOG4FJ6VS	199	EOG42RBTD	156

EOG42BVVN	216	EOG41VHP3	169
EOG41C5GC	215	EOG4DFN6W	419
EOG4K3JFV	353	EOG4RBP48	340
EOG4D7WR0	395	EOG44J13R	256
EOG4D51J1	128	EOG4Q5791	83
EOG4K6DPN	423	EOG4ZKH62	281
EOG4THTD9	231	EOG4ZGMXK	442
EOG4FTTKP	377	EOG4FQZBH	239
EOG4ZGMXV	377	EOG4D51GV	392
EOG4BK3QD	118	EOG4J3V3D	92
EOG4H44NZ	410	EOG46M94F	242
EOG4S7H8Z	227	EOG4JSXQT	417
EOG4ZS7N6	493	EOG4RN8V4	227
EOG41897G	228	EOG4ZW3WB	473
EOG4V15M3	130	EOG48GTNW	332
EOG48KPWS	329	EOG41JWXV	340
EOG437Q17	149	EOG48W9KZ	377
EOG479CSS	282	EOG4QBZQN	327
EOG4NZSD1	246	EOG4W0VZX	276
EOG47PVRM	251	EOG4MSBJ0	301
EOG4K3JH1	62	EOG46Q5BR	308
EOG479CT3	185	EOG4PK0SS	688
EOG4KPRWD	394	EOG49CNSP	618
EOG4VHHRG	487	EOG4N5TH5	123
EOG4MKM1B	481	EOG4THTCM	287
EOG46WWV7	254	EOG4612PB	397
EOG42NGK6	314	EOG4C86CB	180
EOG4WDBX1	421	EOG4JHB1Z	128
EOG4J0ZVF	132	EOG44F4W2	205
EOG4866ZS	178	EOG4B8GZ9	372
EOG4JM68T	224	EOG4QNKF8	656
EOG4KWHBW	476	EOG4FTTJJ	619
EOG4WSTWC	255	EOG45X6FQ	533

EOG4GHX8F	200	EOG41JWXG	348
EOG4M909H	645	EOG4JQ2GH	555
EOG4RV19S	283	EOG4NVX5Q	138
EOG40ZPH0	364	EOG4GQNQQ	309
EOG4CZ92H	78	EOG4NP5P9	228
EOG4X69TT	327	EOG4GQNR6	196
EOG44XH1T	610	EOG4DV45T	348
EOG4C5B3F	553	EOG400048	133
EOG4V6X36	117	EOG4Q5784	266
EOG4ZPCF3	291	EOG4FXPT9	264
EOG437Q0H	291	EOG40ZPH8	270
EOG46T1M3	295	EOG4GHX81	180
EOG4P8D41	151	EOG476HK8	193
EOG48GTP3	228	EOG4ZCRQC	270
EOG4FN333	395	EOG4PRR95	361
EOG4J0ZV8	199	EOG49S4RJ	420
EOG434TRG	322	EOG4GTHZ9	310
EOG4Q83H2	288	EOG4V6X31	147
EOG4PC8BD	338	EOG4KWHC9	364
EOG40GB9P	245	EOG4BRV5B	353
EOG408KT0	649	EOG4N2Z6T	922
EOG4V15M1	205	EOG4QV9XS	198
EOG49GJ2K	237	EOG4MSBJ1	235
EOG4CJT2Q	314	EOG4QZ66C	131
EOG44XH2Q	257	EOG489371	120
EOG4VT4GW	262	EOG42V714	726
EOG47H487	333	EOG4H9W5F	253
EOG45B00H	342	EOG4SQVFV	397
EOG4JQ2GV	360	EOG4GHX70	716
EOG444J5J	197	EOG4X3FKF	513
EOG4DJHG2	348	EOG43TXDM	582
EOG40CG3J	94	EOG43J9PK	744
EOG4DNCQC	207	EOG49322Q	911

EOG4RJDMC	221	EOG45B00C	903
EOG4T4BDW	385	EOG48SFCB	467
EOG4CJT23	321	EOG476HJB	552
EOG4PVMHP	191	EOG49ZW85	650
EOG46DJNF	240	EOG4KH1DV	455
EOG40ZPH3	299	EOG444J47	666
EOG48GTNR	403	EOG4GTHXS	725
EOG434TR7	454	EOG40P2RN	885
EOG4NVX4R	289	EOG43J9PG	1102
EOG480GGX	162	EOG46DJMM	945
EOG4FXPTJ	184	EOG4DR7X7	820
EOG42FR3M	181	EOG4KKWNJ	743
EOG489365	210	EOG48KPWG	330
EOG4RJDMF	242	EOG4H9W5N	196
EOG4X3FKN	363	EOG4WDBXF	290
EOG4S7H8D	358	EOG4NS1XS	173
EOG4PRR9M	193	EOG43TXF5	369
EOG48SFCC	614	EOG4VQ87R	358
EOG4T4BDK	397	EOG47SQZW	515
EOG4PK0TN	204	EOG4GQNQ9	392
EOG4SN06Z	327	EOG4ZW3VZ	799
EOG451C9K	307	EOG4H18DN	799
EOG48PK4G	337	EOG4HX3MK	190
EOG476HJR	373	EOG463XX2	711
EOG4H44PZ	147	EOG4MW6RP	255
EOG4FJ6VQ	203	EOG42BVTH	751
EOG4HHMN7	292	EOG40VT7F	852
EOG470S29	522	EOG4WDBWX	526
EOG4JM68W	187	EOG44F4VT	299
EOG42NGKB	252	EOG4RFJBN	565
EOG44F4VX	300	EOG4FTTKH	447
EOG47M0J4	204	EOG4C5B3Z	344
EOG4FBGCM	311	EOG44B8M6	730

EOG4QBZQJ	399	EOG4SQVFR	755
EOG4T1G6B	259	EOG4PK0ST	431
EOG4C86BS	407	EOG4JWSZP	479
EOG4X3FKR	381	EOG4866XH	660
EOG4S4N1Q	274	EOG47WM6K	748
EOG408KV3	208	EOG46T1KH	640
EOG4PVMJK	195	EOG47D81K	524
EOG4SF7S1	340	EOG42JM9D	567
EOG4V15K0	753	EOG42FR34	673
EOG49S4SD	180	EOG437PZV	757
EOG41896F	529	EOG49S4R4	1635
EOG4S4N17	193	EOG4DV456	698
EOG4PVMJ2	330	EOG4G1K15	1080
EOG476HK3	285	EOG4J9KHW	593
EOG4GQNQ1	658	EOG4PRR8H	1293
EOG43J9QT	188	EOG4R22CK	1585
EOG46Q5BW	384	EOG4RJDK9	1094
EOG4NGF6V	172	EOG4RN8TB	747
EOG4KKWP7	183	EOG4RV197	862
EOG48CZDG	457	EOG4SJ3ZN	1358
EOG4TB2WK	208	EOG4V15JR	1051
EOG4BVQCQ	666	EOG4WM3CP	877
EOG431ZHM	395	EOG46T1KW	338
EOG4QFV0H	146	EOG4T76ND	447
EOG45HQGM	472	EOG47H48J	305
EOG4RN8W3	123	EOG4DBS08	360
EOG4STQPQ	516	EOG4H9W4P	394
EOG4280KK	574	EOG44B8MK	410
EOG4MGQT0	401	EOG46HDVH	862
EOG441NXR	134	EOG43BK87	133
EOG4B2RH7	186	EOG42RBS6	662
EOG4S7H8F	405	EOG4TQJW1	361
EOG4GB5RZ	253	EOG4C86BG	553

EOG466T5W	240	EOG4V9S93	351
EOG415F0F	214	EOG49S4R9	730
EOG447DCV	287	EOG4RR52Q	397
EOG4G4F87	750	EOG470S2X	323
EOG4VQ87G	421	EOG48PK5R	76
EOG4Z616H	631	EOG45QFZQ	288
EOG4WWQ3Z	340	EOG4HT7C9	185
EOG4H44NH	642	EOG402VB4	539
EOG4WPZNB	402	EOG4TMPMD	446
EOG48SFCM	314	EOG4QFV08	198
EOG4FXPSJ	483	EOG4RFJCF	325
EOG4BCC5W	657	EOG4G79HH	502
EOG422849	301	EOG4612PM	403
EOG4GHX7C	430	EOG476HJ9	488
EOG4M37V5	498	EOG4MSBH9	514
EOG4254BR	495	EOG4RBP4W	203
EOG4HX3KF	339	EOG4BRV54	387
EOG4QFTZR	317	EOG48935H	571
EOG473N9J	414	EOG4RXWJS	505
EOG4SQVFH	613	EOG48SFC2	607
EOG42283X	470	EOG44QRKQ	295
EOG44QRJT	704	EOG4C2FVQ	500
EOG4SQVGB	251	EOG45QFZ2	933
EOG4Z6174	394	EOG476HHX	1354
EOG49KD8C	787	EOG4BVQCF	1118
EOG41ZCVW	492	EOG4SN075	339
EOG4C5B3K	530	EOG4SN07Z	214
EOG4NZSCZ	326	EOG4NS1W7	1566
EOG48CZFJ	402	EOG4JWT04	507
EOG44MW9Z	572	EOG4GF1ZX	703
EOG498SM1	133	EOG405QJW	1036
EOG41ZCXD	125	EOG40ZPGC	1528
EOG4GF21C	185	EOG42BVV0	402

EOG47WM7S	199	EOG476HJ4	676
EOG49P8JX	132	EOG4HQC39	721
EOG4547JK	249	EOG42FR30	320
EOG48PK4Z	216	EOG495X9T	546
EOG41JWZ3	241	EOG45TB5Z	756
EOG4GQNQJ	305	EOG47SQZF	2245
EOG479CT2	265	EOG4BNZWN	919
EOG4SQVH1	124	EOG4DFN6C	1062
EOG4HX3M8	213	EOG4FJ6TJ	966
EOG4DJHGW	115	EOG4HMGW1	588
EOG4C2FWV	132	EOG4K6DP0	1236
EOG4VHHSR	108	EOG4N02ZQ	1128
EOG49W11S	113	EOG4QV9WQ	1204
EOG473NB1	185	EOG4RN8T9	859
EOG4SXKZ2	297	EOG4X0KB4	1211
EOG4FXPTT	142	EOG44MWBP	309
EOG4GB5SM	107	EOG42284D	293
EOG46HDX9	122	EOG49P8HR	423
EOG4PVMJ8	294	EOG4S1RSB	437
EOG4Z08R1	669	EOG45DV7S	496
EOG486707	75	EOG4547HN	661
EOG48SFD8	150	EOG4RR53N	170
EOG44QRM3	189	EOG4NCJZ7	307
EOG4MGQT1	307	EOG4QFTZT	325
EOG4GHX7Q	298	EOG4P2NM8	631
EOG4N5THC	139	EOG4JM67H	813
EOG44MW9V	673	EOG4MW6S9	129
EOG4MW6RQ	261	EOG4WM3CX	358
EOG41897C	201	EOG4M0CKD	2085
EOG4SN089	106	EOG41RND4	469
EOG4XD2BC	146	EOG47M0GX	583
EOG48CZF9	221	EOG4RFJBQ	592
EOG4H18FV	207	EOG45MKQ5	612

EOG4NS1XK	179	EOG4C2FVD	688
EOG4MPG8P	313	EOG4XKSSV	280
EOG49P8HT	383	EOG4C2FW3	302
EOG4STQR3	120	EOG4NS1XD	267
EOG4F1VP6	223	EOG408KSV	855
EOG4Q2C13	329	EOG4F7M3R	685
EOG4V6X2Q	184	EOG4BNZWP	547
EOG495XB1	404	EOG4DJHF5	2092
EOG4BZKMJ	630	EOG4TMPKT	986
EOG44XH2N	356	EOG41ZCX0	287
EOG45DV8C	278	EOG46DJMZ	357
EOG48PK5H	142	EOG45HQG6	798
EOG431ZJJ	106	EOG4QJQ76	153
EOG447DDC	172	EOG49GJ1H	781
EOG4HQC4F	211	EOG4CRJHR	2692
EOG4BRV57	194	EOG4P2NN5	359
EOG4D51HB	308	EOG4TB2W2	1132
EOG4R4XPD	90	EOG4MGQSM	435
EOG42Z39F	154	EOG48CZD2	1231
EOG479CS5	381	EOG4HMGVB	579
EOG4GTHZ4	360	EOG4612NS	939
EOG4H18F7	347	EOG4BNZWJ	991
EOG4QFTZV	210	EOG4DFN69	1045
EOG4BG7DZ	578	EOG4FBGBM	1633
EOG476HK6	292	EOG4G79H3	1319
EOG4DFN7R	170	EOG4GMSFR	1079
EOG44J13J	497	EOG4H18DG	2056
EOG4RBP47	410	EOG4JM67D	1052
EOG4K6DQF	141	EOG4MCVJ4	1001
EOG4GB5RK	309	EOG4MKM10	1605
EOG4FN33M	173	EOG4MW6QW	984
EOG4S1RTG	177	EOG4TX99J	1834
EOG45HQHB	206	EOG4WPZNW	352

EOG4W0VZH	433	EOG4RFJCZ	128
EOG4KWHD5	144	EOG4KPRWW	227
EOG49KD96	411	EOG4BCC78	234
EOG47SR0R	197	EOG4F1VPF	85
EOG4F1VN3	676	EOG45X6F8	456
EOG4HMGWP	153	EOG4X69TR	317
EOG4GTJ0C	112	EOG476HJ1	1073
EOG4K3JGS	110	EOG4N2Z6X	473
EOG44XH27	357	EOG4TMPMF	324
EOG4H70WN	580	EOG42Z38T	348
EOG4F4QW2	502	EOG447DD1	232
EOG4BK3NV	605	EOG4GHX80	140
EOG4ZKH5D	442	EOG4HX3JW	852
EOG47WM6Z	676	EOG41NS5Q	375
EOG4MSBHJ	340	EOG4G1K1B	927
EOG480GFH	733	EOG47WM6H	999
EOG4MGQSB	595	EOG4BRV4H	876
EOG4ZGMXD	508	EOG4CRJHV	1088
EOG47SQZM	734	EOG42RBS9	461
EOG49W10Q	362	EOG4GXD5H	1733
EOG4SQVFN	801	EOG48W9M9	372
EOG41ZCVS	936	EOG4QFTZX	250
EOG4D2584	433	EOG4547HH	634
EOG4WDBWQ	808	EOG4HMGV9	599
EOG43N5XG	931	EOG4BZKMM	438
EOG4S4N0W	1167	EOG4BVQCD	1414
EOG4QNKFZ	440	EOG42FR2B	825
EOG4TDZ4B	438	EOG43FFFT	614
EOG44J137	380	EOG4BVQDH	793
EOG470S3P	109	EOG47SQZV	787
EOG4FBGCB	361	EOG4WDBXJ	392
EOG48GTNP	383	EOG4PK0SX	617
EOG4JDFS4	430	EOG4FXPSV	448

EOG45MKQ6	672	EOG40GB8M	1027
EOG4D7WQP	571	EOG44B8M5	753
EOG4DFN6P	490	EOG4D2583	515
EOG4P8D2Z	712	EOG4FFBM1	1212
EOG4V9S8M	564	EOG4RN8TS	301
EOG4V15JZ	433	EOG4RR52H	678
EOG42JM9M	530	EOG4P5HVG	415
EOG4MPG8H	378	EOG466T5J	615
EOG415DZC	568	EOG4JQ2HB	280
EOG476HJ7	532	EOG4G1K18	997
EOG4GB5R8	637	EOG4V6X24	468
EOG415DZ5	1075	EOG4X9628	398
EOG4FQZ9G	943	EOG4J6Q8M	1092
EOG4ZS7N0	1006	EOG43FFFK	2400
EOG4X963K	131	EOG49W107	874
EOG4FXPV6	91	EOG4JHB0H	1127
EOG4JWT03	289	EOG4P2NMH	451
EOG408KTH	359	EOG4Q83G7	1017
EOG4GXD6X	180	EOG4M0CMQ	178
EOG4CNPB6	255	EOG48SFBZ	856
EOG473NB2	241	EOG41G1P2	964
EOG4HQC4T	112	EOG4C2FV7	1240
EOG4X69VH	191	EOG4FJ6TH	2426
EOG466T5H	441	EOG4HHMM3	2022
EOG476HK9	207	EOG4S1RRW	1136
EOG4F1VP2	216	EOG4F1VNH	359
EOG4866Z2	307	EOG41JWXM	351
EOG4T4BF8	284	EOG4S4N1M	346
EOG4N8PQJ	337	EOG4KPRWQ	282
EOG4M90B0	335	EOG43FFGT	220
EOG48KPXZ	202	EOG4K98XS	231
EOG4PK0V1	122	EOG4CC2MF	253
EOG4TTF2V	661	EOG4X0KD1	112

EOG40ZPH5	371	EOG43BK7N	248
EOG48W9MZ	192	EOG4C86C3	369
EOG45QFZ6	604	EOG4BCC6C	377
EOG4TX9BB	314	EOG4PC8C3	185
EOG44F4VM	450	EOG4N8PQ4	389
EOG444J4D	548	EOG4905TZ	587
EOG451C92	409	EOG4KPRX8	113
EOG4FXPSS	352	EOG493241	146
EOG42Z399	225	EOG4RFJCR	150
EOG4MSBHD	415	EOG4XD2BK	105
EOG4KD552	722	EOG4RJDMS	162
EOG4GHX7X	306	EOG4QZ64T	677
EOG44MWB6	466	EOG4P2NMT	295
EOG4B2RHM	126	EOG4NK9F3	463
EOG4N5TGH	288	EOG40CG2C	345
EOG44MWC8	202	EOG49KD9R	156
EOG4N8PQK	293	EOG4WH74S	627
EOG4GQNQV	224	EOG4PC89X	665
EOG4F4QX0	338	EOG4FQZ9S	502
EOG4HT7CC	169	EOG4WWQ44	287
EOG473N9V	321	EOG4PNW1S	605
EOG4DBS0V	184	EOG45X6F0	600
EOG4905VS	211	EOG42BVVR	178
EOG447DC9	443	EOG4P2NN1	308
EOG4GHX74	501	EOG4N5TFX	505
EOG40ZPHF	321	EOG41C5FQ	302
EOG4V6X2J	201	EOG4M642G	722
EOG4BG7GM	133	EOG4GHX7M	505
EOG4JDFT3	211	EOG4C2FVX	341
EOG4FJ6TS	599	EOG4JWSZ6	1197
EOG4TX9B3	422	EOG47D820	252
EOG4MGQSF	462	EOG4J0ZSX	300
EOG4254CR	238	EOG4B5MQ3	712

EOG47PVRQ	216	EOG40VT7J	677
EOG49KD8K	372	EOG4JSXQZ	328
EOG46Q5BV	349	EOG4NVX4Z	340
EOG4PZGS6	229	EOG4P5HVB	550
EOG400038	682	EOG4NK9DS	400
EOG4NK9FZ	132	EOG4TTF32	492
EOG45X6F3	608	EOG44MW9Q	1403
EOG4JDFSJ	252	EOG4MSBHH	497
EOG4JSXRB	407	EOG4612P3	517
EOG4G4F92	233	EOG4W0VZP	352
EOG4866XS	452	EOG447DC4	937
EOG4MKM1R	388	EOG408KSR	1081
EOG473NBF	169	EOG42BVTC	1089
EOG43R25S	498	EOG4J0ZST	1626
EOG4KSN49	300	EOG4JSXQ6	1708
EOG40GBB2	239	EOG4CC2M8	201
EOG4J9KHT	642	EOG4V6X23	345
EOG42NGJJ	481	EOG4RV1BH	177
EOG4BCC5V	816	EOG41896D	522
EOG45B00G	578	EOG4V41TH	285
EOG47M0GT	668	EOG4TMPN0	235
EOG4WH75Q	206	EOG4DZ0FM	117
EOG4T4BDV	343	EOG415F03	201
EOG4DZ0CZ	810	EOG4XKSS9	442
EOG4BRV4R	397	EOG4NVX4W	304
EOG44J13C	533	EOG4280M2	349
EOG46HDW4	497	EOG4BVQCZ	399
EOG4905V7	515	EOG4GB5RX	307
EOG4QBZQ6	646	EOG4C86BD	773
EOG483BPQ	426	EOG4D51H8	383
EOG466T5T	203	EOG4SQVG8	293
EOG4J6Q9J	262	EOG45MKQZ	232
EOG49323P	263	EOG42NGJG	534

EOG4ZCRQ1	295	EOG4QFTZ3	655
EOG4CC2K6	406	EOG4RN8V5	297
EOG4Q5787	232	EOG44QRJR	772
EOG4XGXJV	310	EOG4F1VN9	375
EOG48PK4M	419	EOG4CNP93	503
EOG4NK9DK	575	EOG4WPZMH	761
EOG4HDRCN	423	EOG43FFFR	793
EOG4WSTVK	563	EOG4WSTVW	474
EOG4W3R60	705	EOG4CVDRT	887
EOG49CNSJ	779	EOG45MKQT	421
EOG40P2S2	488	EOG4S7H7V	873
EOG4CZ90Q	644	EOG40VT7C	822
EOG431ZJP	165	EOG4612P4	395
EOG4612Q5	228	EOG4TTF2T	576
EOG4Z617N	177	EOG4QNKF5	628
EOG4ZCRQR	168	EOG4VHHRM	545
EOG4D258S	291	EOG47PVR0	351
EOG495XB5	403	EOG4QFTZ6	931
EOG46WWVF	191	EOG4TB2W7	907
EOG4J9KK6	166	EOG495X9M	998
EOG4M0CN0	270	EOG4612PK	340
EOG479CTV	93	EOG4B5MQT	263
EOG4FN33K	260	EOG42Z382	958
EOG45QFZM	395	EOG4R7SW7	389
EOG4X9622	725	EOG415DZR	357
EOG40VT89	268	EOG4SF7R3	517
EOG4CZ91V	229	EOG42BVTX	541
EOG4905VQ	185	EOG489356	1473
EOG4N2Z85	145	EOG48SFBS	2090
EOG4V6X1Q	577	EOG4GXD5J	1373
EOG4254BM	1002	EOG4XSJ7M	1562
EOG4TB2WN	300	EOG4H70WH	545
EOG4PNW24	417	EOG46DJMJ	743

EOG4H70X6	406	EOG4PVMJ5	306
EOG4TMPNB	144	EOG441NW8	719
EOG469PDM	244	EOG45HQGF	686
EOG4Z08QV	411	EOG4WPZMS	415
EOG47H486	418	EOG4V41SF	1557
EOG43TXDR	429	EOG42V70Z	1521
EOG40K6J9	308	EOG470S38	332
EOG48GTP9	199	EOG4HMGVC	520
EOG4254BT	505	EOG4CVDTC	421
EOG45DV8W	102	EOG45HQHQ	178
EOG49GJ1T	342	EOG4TDZ5J	221
EOG4Q83GM	440	EOG4GB5S9	169
EOG4K0P66	864	EOG4D51J4	193
EOG4CC2K5	746	EOG4WH753	316
EOG4DNCP9	717	EOG4VHHSK	334
EOG4RFJD3	127	EOG49S4RW	299
EOG4P5HW4	217	EOG466T5Z	187
EOG40RZ16	351	EOG463XWX	597
EOG483BPH	672	EOG4QFTZ5	697
EOG4V9S8J	823	EOG4MW6R6	481
EOG4WWQ3D	626	EOG4G79JG	242
EOG4RR52B	859	EOG4JWSZB	765
EOG4GHX78	485	EOG4SJ3ZM	1081
EOG4CFXTT	429	EOG4WM3DG	268
EOG4N02ZS	822	EOG4F7M42	638
EOG4JSXQF	888	EOG44F4TV	1491
EOG47H481	760	EOG4W9GNP	1116
EOG4FTTJK	594	EOG4HQC3C	482
EOG49322M	1146	EOG4SXKXB	1041
EOG4GB5R1	809	EOG4RBP3H	688
EOG4VQ872	1092	EOG4PK0SN	1023
EOG4W9GNM	1631	EOG4J0ZTB	405
EOG4X3FKP	459	EOG4ZS7N4	551

EOG4NP5NG	542	EOG4WSTX5	86
EOG4GF205	519	EOG4WDBX3	369
EOG4CRJJC	247	EOG4QNKFW	269
EOG4ZW3W4	535	EOG4HHMN3	576
EOG408KST	836	EOG44QRKK	470
EOG4280N0	119	EOG463XX1	656
EOG4Q2C0S	408	EOG4MGQS4	1041
EOG4JWSZD	644	EOG42RBS1	1097
EOG4V41SP	467	EOG44J13M	465
EOG4FXPSK	524	EOG4BK3NJ	1782
EOG41896R	523	EOG46Q5C1	336
EOG4VT4G6	532	EOG408KV7	201
EOG4GXD5P	1114	EOG4SJ406	723
EOG45DV78	738	EOG49S4RK	467
EOG4K0P69	484	EOG4ZCRPM	490
EOG4XWDHS	214	EOG463XZD	152
EOG4DZ0DP	853	EOG4GB5R7	606
EOG4ZGMX8	657	EOG4H18FP	566
EOG4QJQ6C	531	EOG4W6MDX	639
EOG4NGF5D	1444	EOG4BNZZ2	170
EOG4KPRVW	588	EOG44J12X	787
EOG40P2RK	980	EOG4S7H7S	1601
EOG42Z386	774	EOG4G79H2	1990
EOG43TXD9	1189	EOG4ZPCDB	502
EOG495X9P	698	EOG4B2RGV	775
EOG4G79H4	1131	EOG44XH1K	1151
EOG4HQC34	746	EOG4280KB	2634
EOG4PVMHD	907	EOG40P2RM	1352
EOG4RFJBC	764	EOG44MWBC	320
EOG4S1RS2	815	EOG44J134	667
EOG4ZS7N2	687	EOG4J3V22	537
EOG4WWQ4H	204	EOG480GFV	940
EOG47WM82	126	EOG43N5ZK	274

EOG4FFBN3	108	EOG469PCR	724
EOG4280M6	314	EOG4H9W48	1032
EOG4J6Q92	543	EOG44MWBT	422
EOG4DV46Q	123	EOG4S4N0V	3073
EOG4K6DPQ	438	EOG49P8J2	691
EOG4K6DPW	340	EOG4SBCGT	1048
